# Sensory primary cilium is a responsive cAMP microdomain in renal epithelia

**DOI:** 10.1038/s41598-019-43002-2

**Published:** 2019-04-25

**Authors:** Rinzhin T. Sherpa, Ashraf M. Mohieldin, Rajasekharreddy Pala, Dagmar Wachten, Rennolds S. Ostrom, Surya M. Nauli

**Affiliations:** 1 Department of Biomedical & Pharmaceutical Sciences, Chapman University, Irvine, CA USA; 2 Institute of Innate Immunity, Department of Biophysical Imaging, University Hospital, University of Bonn, Bonn, Germany; 3 Minerva Max Planck Research Group, Molecular Physiology, Center of Advanced European Studies and Research, Bonn, Germany; 4 Department of Medicine, University of California Irvine, Irvine, CA USA

**Keywords:** Cell signalling, Cellular imaging

## Abstract

Primary cilia are hair-like cellular extensions that sense microenvironmental signals surrounding cells. The role of adenylyl cyclases in ciliary function has been of interest because the product of adenylyl cyclase activity, cAMP, is relevant to cilia-related diseases. In the present study, we show that vasopressin receptor type-2 (V2R) is localized to cilia in kidney epithelial cells. Pharmacologic inhibition of V2R with tolvaptan increases ciliary length and mechanosensory function. Genetic knockdown of V2R, however, does not have any effect on ciliary length, although the effect of tolvaptan on ciliary length is dampened. Our study reveals that tolvaptan may have a cilia-specific effect independent of V2R or verapamil-sensitive calcium channels. Live-imaging of single cilia shows that V2R activation increases cilioplasmic and cytoplasmic cAMP levels, whereas tolvaptan mediates cAMP changes only in a cilia-specific manner. Furthermore, fluid-shear stress decreases cilioplasmic, but not cytoplasmic cAMP levels. Our data indicate that cilioplasmic and cytoplasmic cAMP levels are differentially modulated. We propose that the cilium is a critical sensor acting as a responsive cAMP microcompartment during physiologically relevant stimuli.

## Introduction

The cilium is a dynamic structure that acts as a mechanosensory appendage of the cell and is involved in the pathogenesis of polycystic kidney disease (PKD)^1–4^. The hair-like cilium extends from epithelial lining cells into the lumen and bends upon fluid shear stress, triggering an influx of calcium^3–7^. It has previously been shown that the functional efficiency of the cilia depends on the length; i.e., longer cilia can detect a lower magnitude of shear stress and elicit a larger increase in calcium influx^8–10^. Structural defects of the cilium, such as in intra-flagellar transport protein 88 (IFT88) deletion resulting in shortened cilia, cause ciliopathy phenotype in PKD mouse models^1^. Likewise, functional defects of the cilium, such as dysfunctional polycystins proteins (PC1 or PC2), cause polycystic kidneys^2–4,11,12^. Both PC1 and PC2 form a mechanosensory complex in the cilium, which upon bending modulates calcium fluxes^13,14^.

Similar to calcium, cyclic adenosine monophosphate (cAMP) is a second messenger involved in different signaling pathways. Studies have shown that increasing cAMP levels in cystic epithelial cells, via either adenylyl cyclase (AC) activation or addition of membrane-permeable cAMP analogs, enhances cyst formation and/or cyst enlargement. The cAMP-mediated cystogenic effect has been observed in *in vitro* cultures obtained from murine models and intact cysts excised from PKD patients^15–17^. Arginine vasopressin (AVP), the endogenous antidiuretic hormone, is also observed to aggravate the cystic phenotype as it raises cAMP levels by vasopressin activating receptor type 2 (V2R), a G protein-coupled receptor (GPCR). Activation of V2R induces an intracellular cAMP increase, leading to the insertion of aquaporin 2 (AQP2) into the apical membrane and regulation of body fluid homeostasis^18,19^. Strategies focusing on blocking vasopressin action have shown reduced cystogenesis in murine models of PKD^20–25^. Polycystic Kidney (PCK) rats develop progressive cystic enlargement of the kidneys and hepatic histologic abnormalities that resemble human autosomal dominant PKD^26^. Using this model, Wang *et al*. evaluated renal cyst development in the PCK rats without circulating vasopressin. PCK rats were crossed with Brattleboro (AVP^−/−^) rats and in the resulting PCK AVP^−/−^ rats, renal cAMP levels were lowered along with a marked reduction in renal cysts^23,27^. The promising results of preclinical data have spurred interest in pursuing V2R antagonists as a prospective treatment for PKD^23–25^. Clinical trials have shown that tolvaptan, a V2R antagonist, is effective in decreasing cyst growth and in slowing the decline of renal function^28–32^.

Cyclic AMP is a ubiquitous second messenger, produced by AC activation upon binding of ligands to GPCR coupled to Gs. Thus, cAMP regulates a wide range of cellular processes, ranging from gene regulation to immune function^33^. Previous studies have demonstrated that increasing intracellular cAMP levels, through the addition of forskolin or a cell-permeable cAMP analog, causes an increase in ciliary length of renal epithelia^34,35^. Consistent with this finding, forskolin or a cell-permeable cAMP analog also increases ciliary length in vascular endothelia^36^. Interestingly, forskolin or a cell-permeable cAMP has a minimal effect in serum-deprived synoviocytes^37^. Furthermore, inhibition of ciliary-localized adenylyl cyclase 3 (AC3) induces primary cilia elongation in synoviocytes^37^. However, the effect of tolvaptan in modulating ciliary length through cAMP has never been evaluated. Moreover, the dynamic level of cilioplasmic and cytoplasmic cAMP has not been previously examined with a targeted sensor. Given the potential treatment of tolvaptan and the role of cilia in PKD, we sought to characterize the effect of tolvaptan on ciliary length and ciliary cAMP signaling.

We show that tolvaptan increases ciliary length and enhances mechanosensitivity in response to shear stress. We also show that V2R and AC3 are localized in renal epithelial cilia. Monitoring cAMP levels in the cilioplasm after stimulation with tolvaptan revealed that the cilium is a distinct cAMP microdomain. Changes in cilioplasmic cAMP levels were found to be distinct and independent from cytoplasmic level of cAMP. Furthermore, shear stress decreased cilioplasmic, but not cytoplasmic cAMP below basal levels. We therefore hypothesize that primary cilia are chemosensitive and mechanosensitive organelles that form dynamic cAMP microdomain distinct from cell body.

## Results

### Tolvaptan increases ciliary length and enhances shear-stress induced cytosolic calcium increase

**Figure 1:**
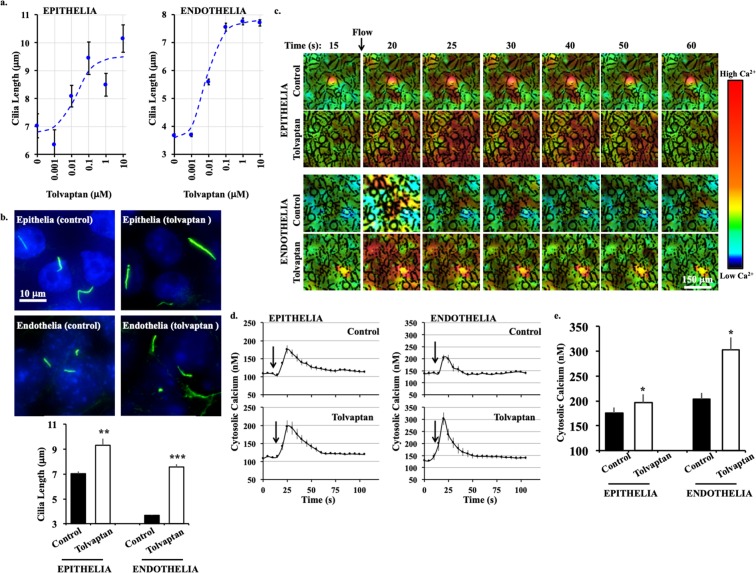
Tolvaptan increases cilia length and enhances mechanosensitivity. (**a)** Dose-response relationship between different concentrations of tolvaptan and ciliary length is shown in the Sigmoidal plot. Distribution of ciliary length for individual tolvaptan concentration is shown in Supplementary Fig. S1. (**b**) Representative immunostaining images of epithelial and endothelial cells treated without (control, vehicle) and with tolvaptan (0.1 μM) for 24 hours. Cilia (acetylated-α-tubulin) are shown in green and nucleus in blue. Corresponding bar graphs show ciliary length averages. (**c**) Time-lapse images represent the intracellular calcium level in response to fluid-shear stress (arrow) by epithelial and endothelial cells treated without (control, vehicle) and with tolvaptan (0.1 μM). Color bar indicates intracellular calcium level from low (black) to high (red). Corresponding brightfield images are shown in Supplementary Fig. S1. (**d**) Averaged intracellular calcium levels are plotted across time in second (s). Arrows indicate the start of fluid-shear stress. (**e**) Bar graph shows averaged intracellular calcium peak induced by fluid-shear. N = 3 experiments for each group. For each N, a minimum of 50 cells were analyzed. *P < 0.05, **P < 0.001, and ***P < 0.0001 compared to the corresponding control groups.

To determine if tolvaptan could alter the ciliary length, we measured the ciliary length distribution in epithelial and endothelial cells after treatment with different concentrations of tolvaptan (Supplementary Fig. S1). Tolvaptan-treatment resulted in a dose-dependent increase in ciliary length in renal epithelial cells. The effect was maximal at 0.1 μM and reached a steady-state at higher concentrations (Fig. 1a,b). In renal epithelial cells, average ciliary length was 7.05 ± 0.15 μm. When treated with 0.1 μM tolvaptan, ciliary length increased to 9.34 ± 0.51 μm. In vascular endothelial cells, which generally have shorter cilia than epithelial cells, ciliary length increased from 3.67 ± 0.06 μm to 7.56 ± 0.14 μm after treatment with tolvaptan.

Both epithelial and endothelial cilia function as mechanosensory organelles by increasing intracellular calcium in response to fluid-shear stress^4–7^. To test whether the increase in ciliary length translated to enhanced mechanosensitivity, cytosolic calcium levels in response to shear stress were measured with Fura-2 (Fig. 1c; Supplementary Fig. S1). After baseline measurement, cells were subjected to shear stress. Pre-treatment with tolvaptan significantly enhanced the calcium response to fluid flow in both epithelial and endothelial cells (Fig. 1d,e). These results show that tolvaptan increases ciliary length and enhances cilia mechanosensitivity.

The cilia are also intertwined with the cell cycle and are usually resorbed during cell division^38,39^. We therefore examined if tolvaptan affected cell division using labeling with propidium iodide, a dye that binds stoichiometrically to DNA, to approximate the distribution of cells at different cell division stages. However, tolvaptan did not affect cell division stages in either epithelial or endothelial cells (Supplementary Fig. S1).

### Vasopressin receptor knockdown modulates ciliary length and function

**Figure 2:**
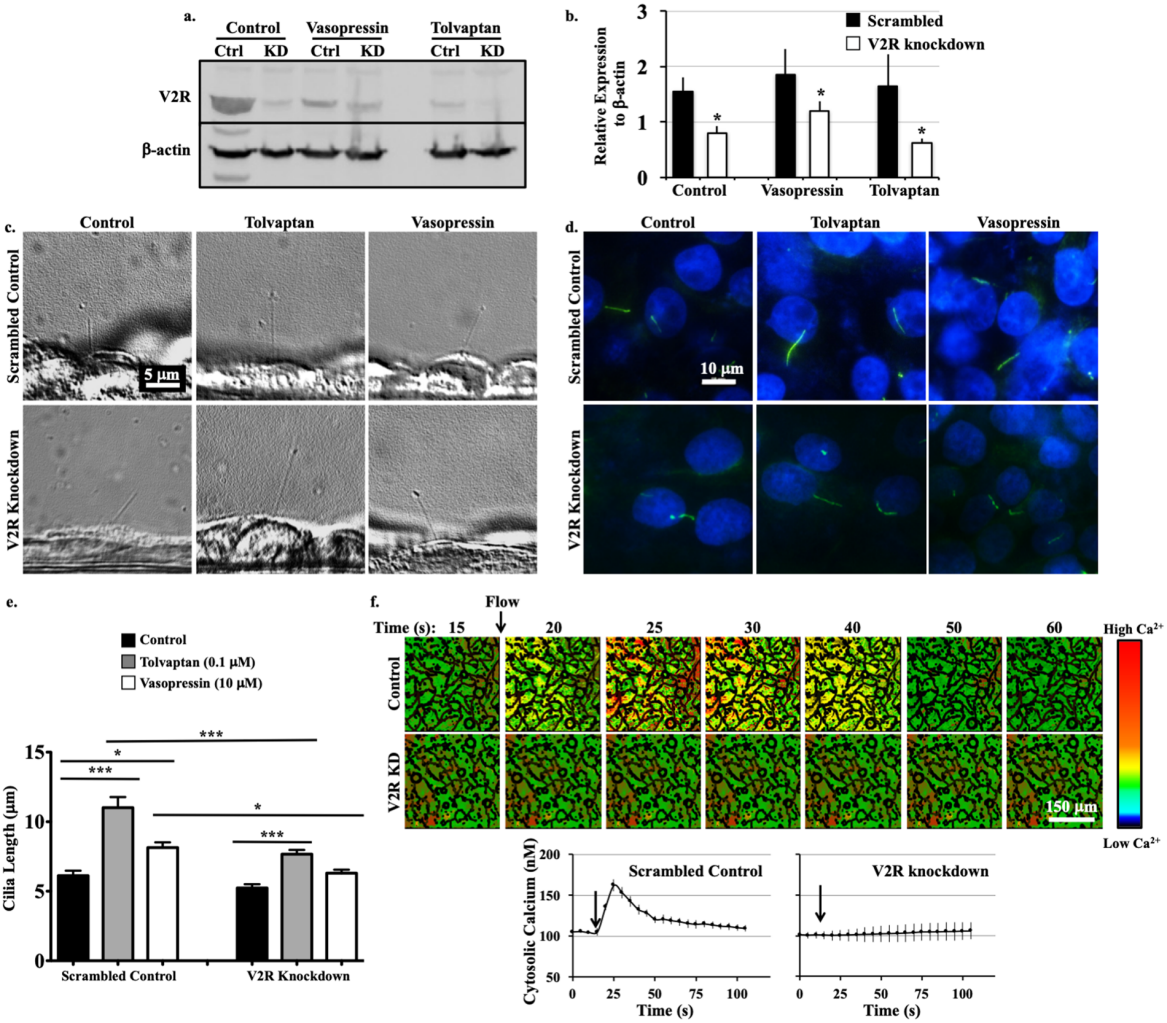
V2R modulates ciliary length and function. (**a**) Representative western blot image of scrambled control (Ctrl) and V2R-knockdown (KD) cells with vehicle (control), vasopressin (10 μM) and tolvaptan (0.1 μM) treatments. (**b)** Normalized V2R expression level is shown. Changes in ciliary length are observed from the phase contrast (side view, **c**) and immunostaining (top view, **d**) images. (**e)** Ciliary lengths are averaged. (**f)** Time-lapse images represent intracellular calcium level in response to fluid-shear stress. Color bar indicates intracellular calcium level from low (black) to high (red). Averaged intracellular calcium levels are plotted across time in second (s). Arrows indicate the start of fluid-shear stress. Corresponding brightfield images are shown in Supplementary Fig. S2. N = 3 experiments for each group. For each N, a minimum of 50 cells were analyzed. *P < 0.05, **P < 0.001, and ***P < 0.0001 compared to the corresponding control groups.

To further validate our results, we generated V2R-knockdown LL-CPK1 epithelial cells using lentiviral transfection. It has been reported that receptor activation or inhibition promotes receptor down-regulation^38,39^; thus, V2R expression was examined in cells with or without vasopressin (10 μM) or tolvaptan (0.1 μM) treatment for 24 hours (Fig. 2a; Supplementary Fig. S2). V2R expression was compared between V2R-knockdown cells and the corresponding scrambled control cells (Fig. 2b). Ciliary length was determined in scrambled or V2R-knockdown treated cells in combination with addition of saline (control), tolvaptan, or vasopressin in live (Fig. 2c) and fixed (Fig. 2d) cells. Cilia in V2R-knockdown cells (5.23 ± 0.27 μm) appeared shorter than in scrambled control cells (6.11 ± 0.36 μm), but the difference was not significant (Fig. 2e). Tolvaptan treatment caused a significant increase in ciliary length of both control cells (11.01 ± 0.76 μm) and V2R-knockdown cells (7.67 ± 0.30 μm). However, the increase was significantly less in the V2R-knockdown compared to controls. Cells treated with vasopression displayed a significant increase in ciliary length in scrambled control cells (8.13 ± 0.38 μm vs. 6.11 ± 0.36 μm in control), but not in V2R-knockdown cells (6.30 ± 0.25 μm vs. 5.23 ± 0.27 μm in V2R-knockdown control).

To investigate the role of V2R-knockdown in fluid-sensing, calcium imaging in response to fluid flow was performed. Although the differences in ciliary length were negligible between the V2R-knockdown and control cells, the V2R-knockdown failed to show the characteristic peak of calcium influx upon fluid flow (Fig. 2f; Supplementary Fig. S2). Previous work has also shown that the V2R is linked to calcium mobilization in rat IMCD cells, where activation of the V2 receptor results in a transient calcium increase, although the underlying mechanism has not been established^40^.

**Figure 3:**
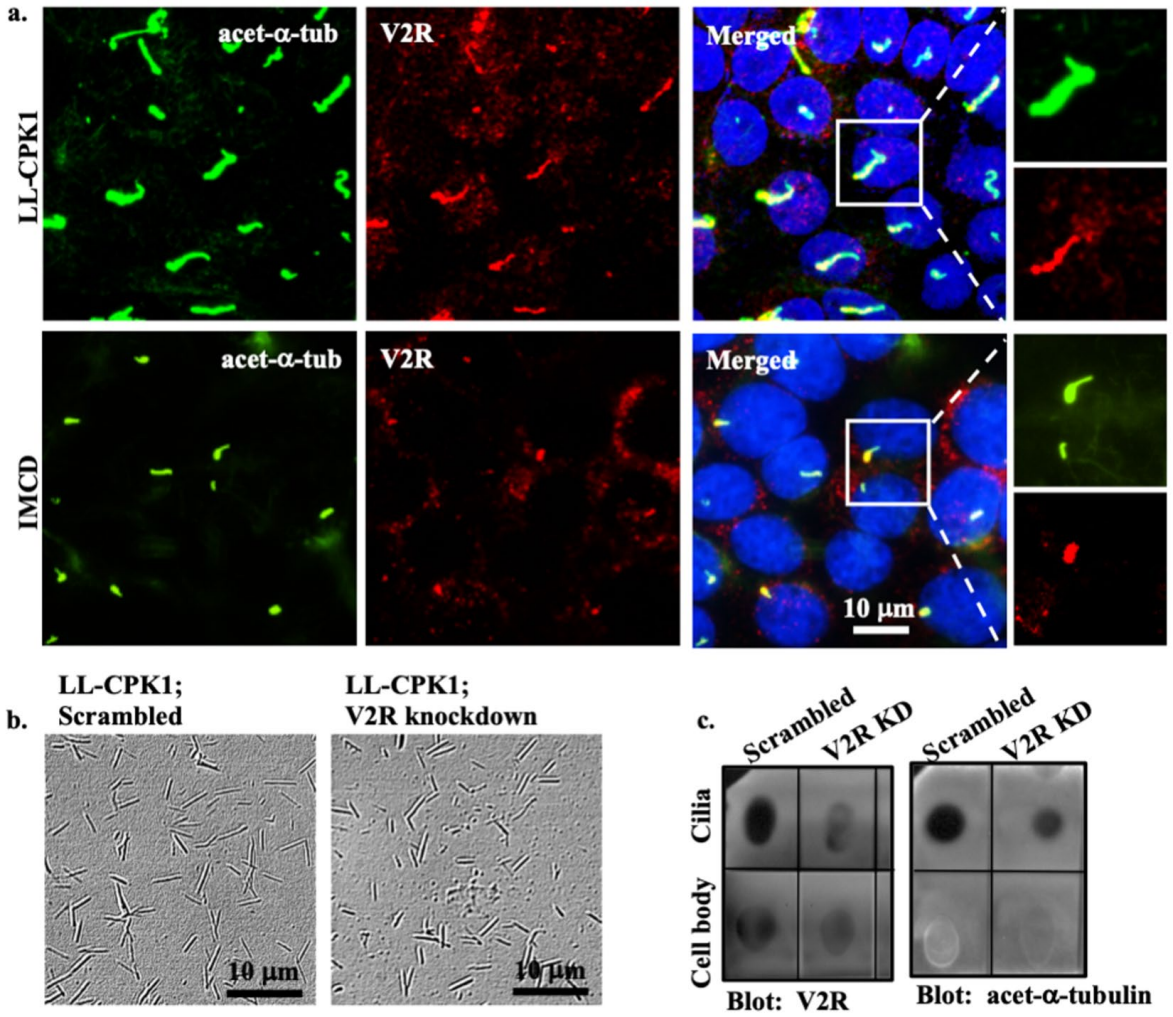
V2R is localized to primary cilia. (**a)** Confocal images of renal epithelial from pig (LL-CPK1) and dog (IMCD) show localization of V2R (red) at the base of cilia. Acetylated-α-tubulin (acet-α-tub) used as ciliary marker is shown in green and nucleus in blue. Maximum intensity projection images from accumulated z-stack for LL-CPK1 (scrambled and V2R-knockdown), IMCD and endothelial cells are shown in Supplementary Figs S3 and S4. (**b)** Phase contrast images represent isolated cilia from scrambled control and V2R-knockdown LL-CPK1 cells. (**c)** Dot blot indicates the presence of V2R in both isolated-cilia and cell-body extracts. Isolated cilia extracts are confirmed by the presence of acetylated-α-tubulin.

Next, immunolocalization was performed to reveal whether V2R localizes to primary cilia (Fig. 3a). IMCD cells, which have been shown to contain V2R in the cilium, were used as a positive control^41,42^. In LL-CPK1, V2R localization was observed throughout the cilium and in IMCD cilium V2R was detected in the base of cilium (Fig. 3a; Supplementary Fig. S3). V2R-knockdown cells showed very little membrane localization of V2R (Fig. S3). Because most endothelial cells did not show V2R localization to primary cilia (Supplementary Fig. S3), we only used LL-CPK1 in the remainder of our studies. Additionally, LL-CPK1 cells were grown on permeable supports and then used for immunolocalization. After scanning Z-stack images, XZ and XY planes were reconstructed which showed similar distribution of V2R in the apical and basolateral membrane (Supplementary Fig. S4).

To further confirm the presence of V2R in the cilia, primary cilia were isolated using shear stress^43,44^. Isolated cilia from scrambled and V2R-knockdown cells were verified with a brightfield microscope (Fig. 3b). Due to a low concentration of total protein collected in the isolated cilia extract, a dot blot was used (Fig. 3c). The acetylated-α-tubulin blot was to indicate the purity of our cilia lysate and the corresponding V2R blot established that the V2R was associated with the cilia.

### Primary cilia are cAMP responsive microdomains

**Figure 4:**
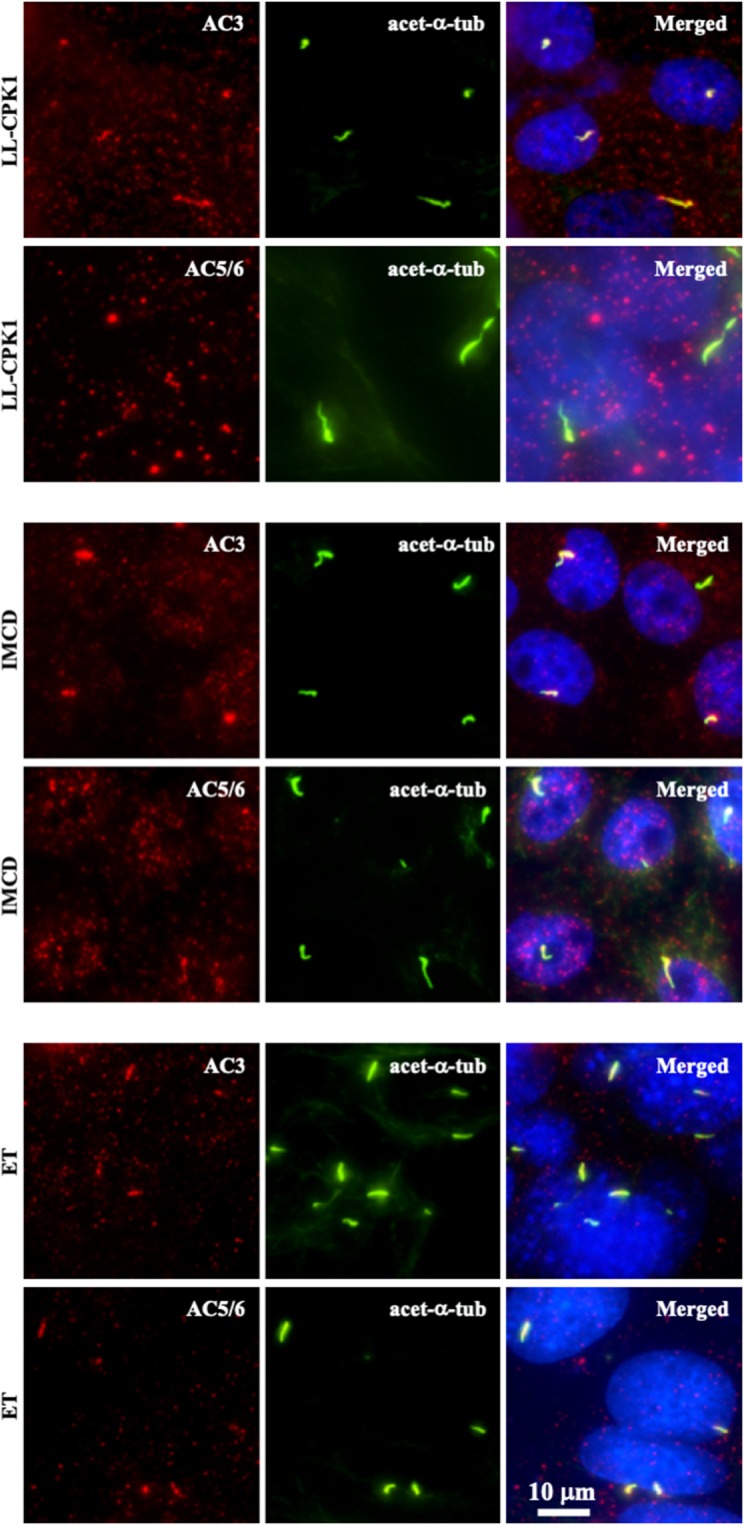
Adenylyl cyclase isoforms are differentially expressed to cilia in different cell lines. Adenylyl cyclase-3 (AC3; red) is localized to cilia in renal epithelial (LL-CPK1 and IMCD) and vascular endothelial (ET) cells (Supplementary Fig. S5 for an additional LL-CPK1 image). Adenylyl cyclase-5/6 (AC5/6; red) is localized to cilia in IMCD and ET cells, but not in LL-CPK1. Acetylated-α-tubulin (acet-α-tub) used as ciliary marker is shown in green and nucleus in blue. Additional images for other AC isoforms are shown in Supplementary Fig. S5.

V2R is a Gs-coupled GPCR, which activates AC and, thereby, increases intracellular cAMP levels^45,46^. AC3 has been reported to localize to neuronal primary cilia^47,48^, whereas AC5/6 are expressed in primary cilia of mouse renal epithelial and LL-CPK1 cells^49,50^. We analyzed AC expression in different cell lines and observed a differential expression pattern between the different AC isoforms (Supplementary Fig. S5). AC5/6 was not localized in cilia of LL-CPK1 cells, although it was observed in IMCD and endothelial cilia (Fig. 4). In contrast, AC3 was localized in cilia of LL-CPK1, IMCD, and endothelial cells (Fig. 4; Supplementary Fig. S5).

Because cAMP measurement has traditionally been performed with a solid-phase enzyme immunoassay, we used this technique to measure cAMP levels after tolvaptan treatment or induction of fluid-flow. Neither fluid flow nor tolvaptan treatment changed the cAMP levels, although a forskolin response was observed in both scrambled and V2R-knockdown cells (Supplementary Fig. S6). However, an immunoassay does not allow us to distinguish cilioplasmic from cytoplasmic cAMP changes. To monitor changes in ciliary cAMP levels, we used the ciliary-targeted cAMP sensor 5HT6-mCherry-cADDis. We first confirmed that the kinetics between cilia-specific (5HT6-mCherry-cADDis) and cytosolic (cAMP-cADDis) reporters did not alter (Supplementary Fig. S6).

**Figure 5:**
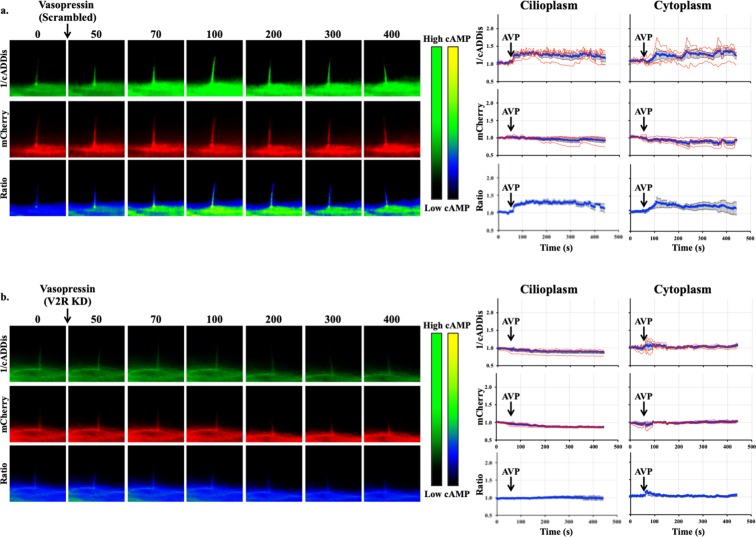
Vasopressin mediates cAMP signaling in cilia. Representative time-lapse images of 1/cADDis, mCherry channels and Ratio (mCherry/cADDis) with their corresponding line graphs showing cAMP levels in response to vasopressin (AVP) in (**a)** scrambled and (**b)** V2R-knockdown cells. Color bar on the left indicates cAMP level from low (black) to high (green) for 1/cADDis channel. cAMP level from low (black) to high (yellow) are shown on the right color bar for the ratio images. Corresponding individual raw traces (red) and average signal (blue) are plotted in line graphs for 1/cADDis, mCherry and ratio signals in the cilioplasm and cytoplasm. Arrows indicate stimuli. N = 4 for each group.

**Figure 6:**
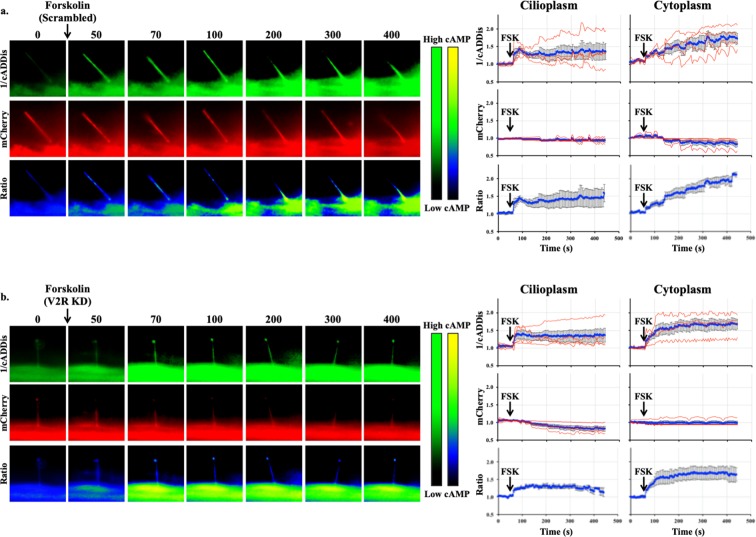
Forskolin increases cAMP level in cilioplasm and cytoplasm. Representative time-lapse images of 1/cADDis, mCherry channels and Ratio (mCherry/cADDis) with their corresponding line graphs showing cAMP levels in response to forskolin (FSK) in (**a)** scrambled and (**b)** V2R-knockdown cells. Color bar on the left indicates cAMP level from low (black) to high (green) for 1/cADDis channel. cAMP level from low (black) to high (yellow) are shown on the right color bar for the ratio images. Corresponding individual raw traces (red) and average signal (blue) are plotted in line graphs for 1/cADDis, mCherry and ratio signals in the cilioplasm and cytoplasm. Arrows indicate stimuli. N = 4 for each group.

**Figure 7:**
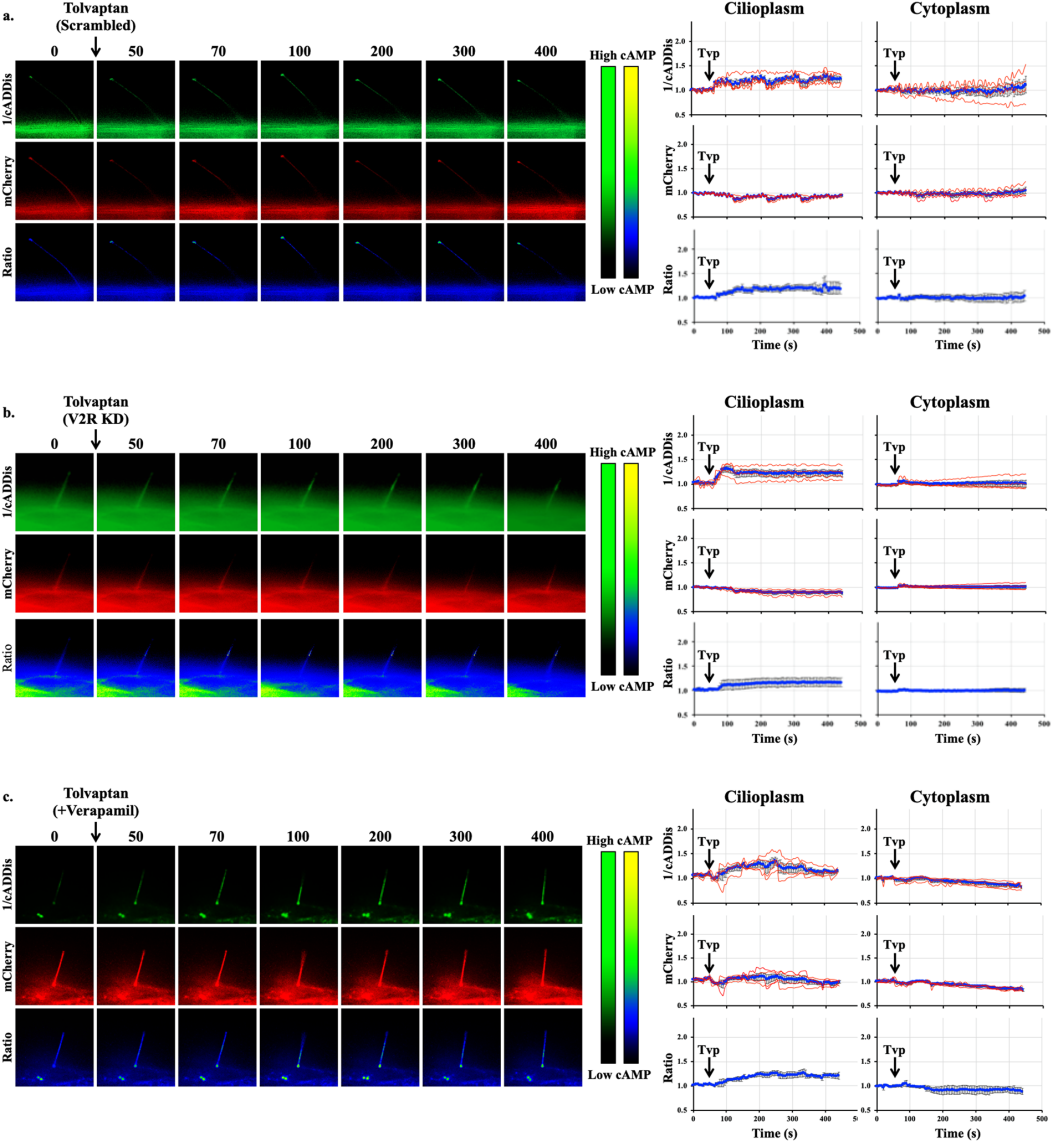
Tolvaptan increases cilioplasmic cAMP. Representative time-lapse images of 1/cADDis, mCherry channels and Ratio (mCherry/cADDis) with their corresponding line graphs showing cAMP levels in response to tolvaptan (Tvp) in (**a)** scrambled, (**b)** V2R-knockdown, and (**c)** verapamil-treated cells. Color bar on the left indicates cAMP level from low (black) to high (green) for 1/cADDis channel. cAMP level from low (black) to high (yellow) are shown on the right color bar for the ratio images. Corresponding individual raw traces (red) and average signal (blue) are plotted in line graphs for 1/cADDis, mCherry and ratio signals in the cilioplasm and cytoplasm. Arrows indicate stimuli. N = 4 for each group.

**Figure 8:**
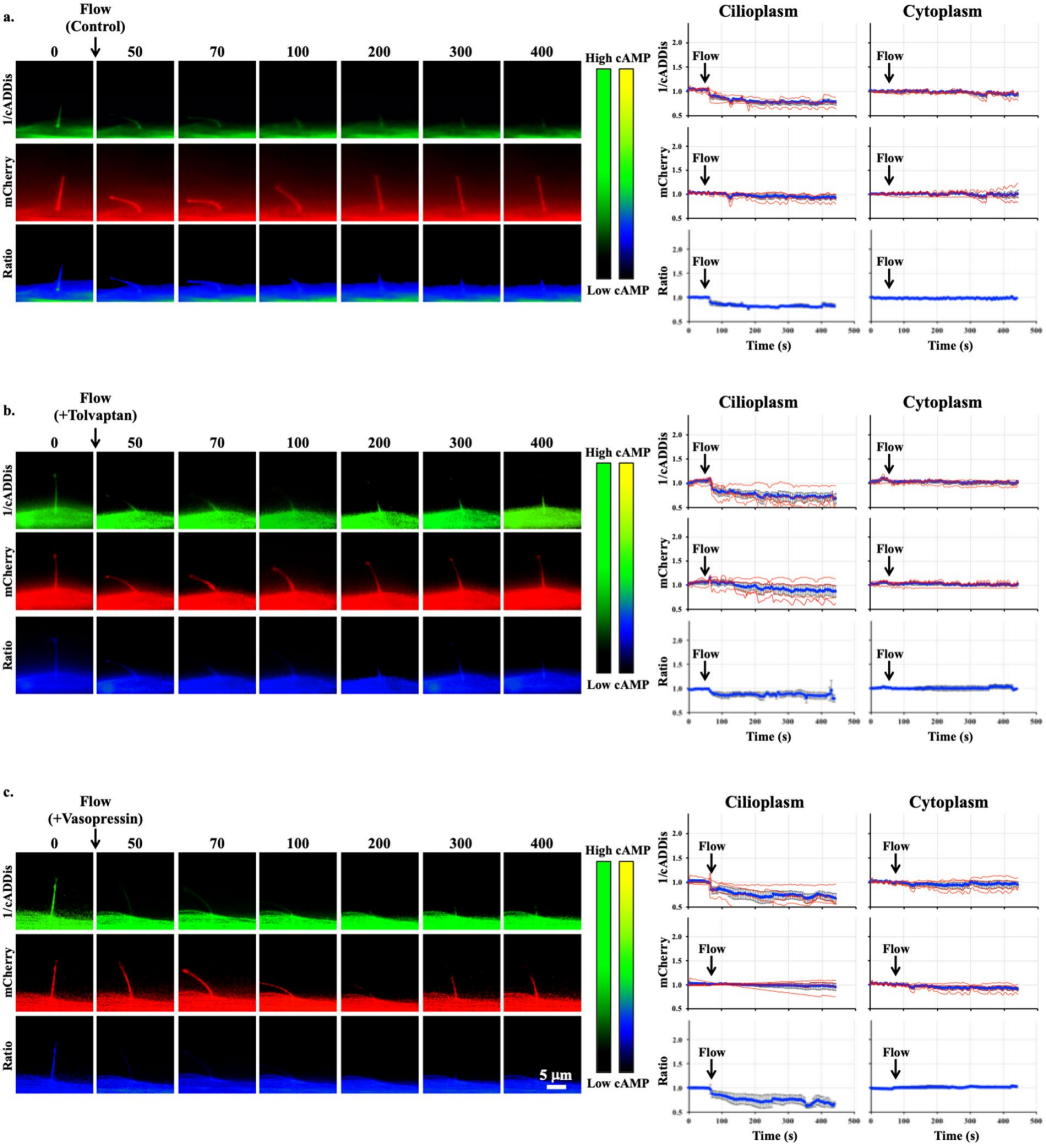
Flow decreases cilioplasmic cAMP level. Representative time-lapse images of 1/cADDis, mCherry channels and Ratio (mCherry/cADDis) in response to fluid-flow (1.0 dyne/cm^2^) after treatment with (**a)** vehicle control, (**b)** 0.1 μM tolvaptan (Tvp) or (**c)** 10 μM vasopressin (AVP). Color bar on the left indicates cAMP level from low (black) to high (green) for 1/cADDis channel. cAMP level from low (black) to high (yellow) are shown on the right color bar for the ratio images. Corresponding individual raw traces (red) and average signal (blue) are plotted in line graphs for 1/cADDis, mCherry and ratio signals in the cilioplasm and cytoplasm. Arrows indicate stimuli. N = 4 for each group.

**Figure 9:**
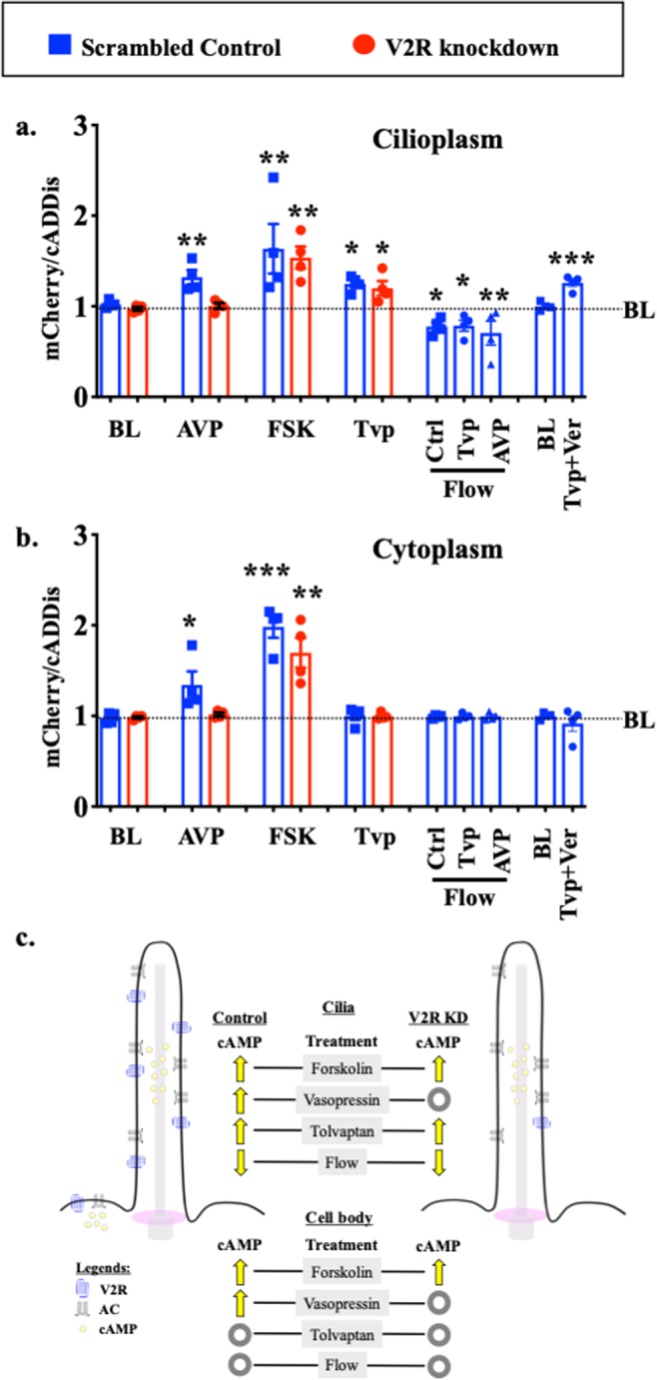
Cilioplasmic and cytoplasmic cAMP levels are differentially regulated. Peak cAMP increase in response to each stimulus is summarized in the bar graph for (**a)** cilioplasm and (**b)** cytoplasm. N = 4 experiments for each group. *P < 0.05, **P < 0.001, and ***P < 0.0001 compared to the baseline (BL) level prior to stimulus; AVP = arginine vasopressin; FSK = forskolin; Tvp = tolvaptan; Ctrl = control; Ver = verapamil. (**c)** Vasopressin receptor type-2 (V2R) is localized to primary cilia in renal epithelia, and V2R activation with vasopressin increases adenylyl cyclase (AC) activity. Tolvaptan but not vasopressin elicits a cAMP increase in V2R-knockdown in primary cilium. Unlike tolvaptan which shows cilia-specific cAMP response, AC activator (forskolin) and vasopressin elicit cAMP responses in both cilioplasm and cytoplasm. Cilioplasmic but not cytoplasmic cAMP signaling is repressed below basal levels when cilia bend by fluid-flow. These responses indicate that a cilium may function as a distinct cAMP microdomain, independent from cytoplasmic cAMP.

Using imaging setup^51^, we were able to monitor both changes in the cytoplasmic and cilioplasmic cAMP levels. Vasopressin (10 μM) elicited an increase in cAMP levels in the cilium and the cell body (Fig. 5a; Supplementary Movie S1). In the V2R-knockdown cells, this response was absent in both the cytoplasm and the cilioplasm (Fig. 5b). Forskolin (5 μM) also increased cAMP levels in the cytoplasm and cilioplasm of scrambled control and V2R-knockdown cells (Fig. 6; Supplementary Movies S2 and S3). Tolvaptan (0.1 μM) induced an increase cilioplasmic cAMP levels, but no significant change was observed in the cytoplasm (Fig. 7a). Interestingly, tolvaptan also increased cilioplasmic cAMP in the V2R-knockdown cells (Fig. 7b). To exclude potential involvement of extracellular calcium influx and the roles of calcium-regulated ACs, cells were preincubated with calcium channel blocker verapamil (2 μM) before being challenged with tolvaptan. Blocking verapamil-sensitive calcium channels had no effect on ciliary cAMP increase nor impacted tolvaptan-induced ciliary length increase (Fig. 7c, Supplementary Fig. S7). In response to flow, the cilioplasmic cAMP underwent a significant decrease beyond the basal level (Fig. 8a; Supplementary Movie S4). The same response was not observed in the cytoplasm, where the cAMP remained at the basal level. Neither vasopressin (10 μM) nor tolvaptan (0.1 μM) affected the flow-induced repression of cilioplasmic cAMP (Fig. 8b,c). In the cytoplasm, cAMP levels remained stable during flow. Taken together, certain stimuli modulated cAMP response differentially between cilioplasm and cytoplasm (Fig. 9), which supports the idea of the cilium being a distinct cAMP microdomain.

## Discussion

Activation of V2R induces a GPCR signaling cascade, resulting in an intracellular cAMP increase. In turn, cAMP-dependent protein kinase (PKA) and other downstream signaling molecules are activated^52,53^. Ultimately, this leads to the insertion of AQP2 into the apical membrane^54^, increasing water reabsorption in the kidney collecting duct and enhancing urinary concentration in the thick ascending limb. Thus, the overall V2R function is to regulate body fluid homeostasis^18^.

Using PCK rats (orthologous to human PKD)^26^ crossed with Brattleboro rats (without circulating vasopressin)^27^, Wang *et al*. found that vasopressin is a powerful modulator of cystogenesis^23^. The PCK AVP^−/−^ rats have lower renal cAMP level and show a marked reduction in renal cysts. Likewise, other animal studies show that inhibition of vasopressin-mediated signaling protects against cystogenesis^24,25^. The preclinical results support the idea of pursuing V2R antagonists like tolvaptan as a prospective treatment for PKD. Clinical data have also shown positive results with tolvaptan significantly reducing the rate of total kidney volume increase and slowing the estimated glomerular filtration rate (eGFR) decline in patients^28–32^. In April 2018, tolvaptan, under the trade name Jynarque was approved by the FDA to slow kidney function decline in adults with PKD. As a note, tolvaptan has been previously approved in 2008 by the FDA, marketed as Samsca, for treatment and prevention of hyponatremia^55,56^. Due to the potential of liver injury caused by tolvaptan, there is an FDA approved Risk Evaluation and Mitigation Strategy (REMS) program that needs to be followed when tolvaptan is prescribed to PKD patients^57,58^. The REMS include requirement of frequent tests for blood bilirubin-total (BT), alanine and aspartate aminotransferases (ALT and AST) levels to evaluate liver toxicity. Discontinuation of treatment can mitigate the hepatotoxic effects of tolvaptan when caught early hence emphasizing the requirement of frequent testing of liver function biomarkers^57,59^.

In our study, we examined the effect of tolvaptan on ciliary function because defects in ciliary function have been associated with PKD. Ciliary dysfunction that prevents sensation of fluid-flow in renal epithelia i.e. calcium fluxes generated in response to fluid-flow, contributes to cyst formation^6,60^. Kidney-specific inactivation of Kif3a, a ciliogenic gene, in newborn mice resulted in the loss of primary cilia and produced kidney cysts^61^. The loss of cilia resulted in aberrant planar cell polarity as measured by the orientation of mitotic spindles in relation to the longitudinal axis of the collecting ducts. When complemented with increased rates of cell proliferation, like in cases of acute renal injury, the aberrant planar cell polarity randomizes orientation of cell division leading to tubular dilatation and kidney cyst formation^62^. Previous studies have shown that the ability of cilia to sense fluid-flow correlates with the ciliary length^63^. Upon treatment with tolvaptan, ciliary length was increased in both epithelial and endothelial cells. In turn, the mechanosensitivity of the cells was increased, as shown by cytosolic calcium measurements in response to fluid-flow. Endothelial cells displayed a more pronounced amplification of the calcium signal, which could be attributed to the more pronounced increase of ciliary length after tolvaptan treatment compared to epithelial cells.

Tolvaptan is a V2R antagonist, thus a V2R-knockdown would be expected to have similar effects as tolvaptan treatment. However, our results indicate that tolvaptan increases ciliary length, whereas V2R-knockdown does not affect ciliary length. Furthermore, tolvaptan also increases ciliary length in V2R-knockdown cells. This could be due to the incomplete deletion of V2R, which allows tolvaptan to act through V2R albeit at a lower magnitude, or the action of tolvaptan is independent of V2R. To this end, we analyzed localization of V2R receptors and observed similar V2R presence in apical and basolateral membranes. We see V2R expressed throughout the cilium in LL-CPK1, as also shown by Raychowdhury *et al*.^49^, and in the basal bodies of primary cilia in IMCD renal epithelial cells. The exact mechanism by which tolvaptan increases ciliary length and function is not clear at present. However, our data indicate that such a mechanism very likely involves ciliary cAMP. The most recent study indicates that tolvaptan can inhibit potassium (K^+^) channels^64^. Thus, the potential of multiple targets or mechanisms of action of tolvaptan remains a possibility.

We performed functional studies to compare cAMP levels in V2R-knockdown cells in response to V2R agonist and antagonist. In our cilia imaging experiments, vasopressin, forskolin and tolvaptan increased cilioplasmic cAMP levels. Vasopressin stimulated cilioplasmic cAMP increases were absent in V2R-knockdown cells, whereas tolvaptan elicited a cilioplasmic cAMP increase independent of V2R. We propose that while vasopressin requires the presence of V2R to engage signaling in the cilium, tolvaptan acts in a V2R-independent manner on ciliary signaling.

Previous studies have shown that the ciliary V2R is coupled with a functional AC that increases cAMP levels in cilia upon vasopressin binding^49^. This is particularly interesting since localized cAMP responses could regulate ciliary function without stimulating global increases in cAMP that would affect cell proliferation in cystic epithelia. V2R activation stimulates AC activity and studies have shown AC isoform 5/6 to be present in the cilia^49,50^. To verify this and discover if any other isoforms are present in the cilium, we immunostained for all AC isoforms. To our surprise we found AC3 in the cilia of LL-CPK1 cells, but there was no cilia localization of AC5/6 as previously reported^49,50^. Of note is that AC3 localization has been reported in neuronal cilia^47,48^. AC3 is one of the three CaM-sensitive AC isoforms although the effect is not as pronounced as seen in CaM activation of AC1 and AC8. Further, it is likely that AC3 is conditionally activated by calcium/calmodulin (CaM) and also by PKC but inhibited by calcium/calmodulin-dependent protein kinase II (CaMK-II)^65–67^. Studies have shown CaM localization at the basal body as well as throughout the cilia in a punctate pattern^68^. In a 2011 study, Rothschild *et al*. showed that CaMK-II is present in zebrafish cilia^69^. Morpholino mediated suppression of CaMK-II in zebrafish embryos induced hydrocephaly and pronephric cysts, and destabilized cloacal cilia. Furthermore, they establish that PKD2 and CaMK-II deficiencies are synergistic, where CaMK-II is a crucial effector of PKD2 Ca^2+^ required for morphogenesis of the pronephric kidney and stabilization of primary cloacal cilia. This suggests that CaMK-II might play a key role in ciliary function and response to physiological stimuli.

Cyclic AMP signaling has the potential to regulate various pathways and cell functions but when restricted in a compartmentalization model, cAMP fluxes can result in specific responses. Based on our cAMP measurements, we noted that changes in cilioplasmic cAMP is independent from cytoplasm. We also found that while vasopressin and forskolin increase both cilioplasmic and cytoplasmic cAMP, tolvaptan and fluid-flow result in a discrete change only in cilioplasmic cAMP. In response to flow, the cilioplasmic cAMP underwent a significant decrease beyond basal levels. Pre-treatment with either vasopressin or tolvaptan does not affect the flow-induced cAMP decrease in the cilia, indicating the complexity of cAMP signaling within cilioplasm. These results support the idea that the cAMP signaling occurs in a compartmentalized manner such that numerous signaling pathways can utilize this biochemical signal and still achieve distinct cellular effects (Fig. 9).

The concept of cAMP being synthesized in specific microdomains and spatially regulated is of growing interest. Studies with membrane microdomain specific sensors show that in addition to the local phosphodiesterases, which control the levels of cAMP, there are specific arrangements of the cAMP signaling components^70–73^. Scaffold proteins, such as A-kinase anchoring proteins (AKAPs) and perhaps others, bring together GPCR, AC isoforms, phosphodiesterases and protein kinase A to create signaling complexes that regulate spatially constricted cAMP signaling cascades. The small cilia organelle enables enrichment of proteins with respect to the rest of the plasma membrane and may also restrict diffusion of second messengers with a transition zone that separates the cilioplasm from the cytoplasm^74–78^. These features make the cilia an effective signaling microdomain with the ability to generate a high local concentration of second messengers and effectors proteins. Studies have shown the presence of GPCR, AC isoforms and cAMP-mediated pathways present in the cilium^49,79^. It is plausible to contemplate that cAMP could play a role in the dynamic modulation of the cilia, which is linked to its functional efficiency.

In conclusion, we find that tolvaptan can modulate ciliary length and function. Interestingly, tolvaptan does not fit the mold of a traditional antagonist, at least not within the ciliary structure. The prospect of tolvaptan working through an off-target effect in the cilia might open new strategies targeting the cilia and PKD relationship. In studying effects V2R/tolvaptan in cilioplasmic cAMP, our data further establish the cilium as a cAMP microdomain that responds to physiologically relevant stimulus.

## Materials and Methods

### Reagents and antibodies

For cell culture, Dulbecco’s Modified Eagle Medium (DMEM), trypsin, penicillin-streptomycin solution, phosphate buffered saline (PBS), Dulbecco’s Phosphate-Buffered Saline (DPBS) and were acquired from *Corning* (Manassas, VA). Fetal bovine serum (FBS) was obtained from *Seradigm* and Dulbecco’s phosphate buffered saline (DPBS) from *HyClone* (Logan, UT). Sucrose, Triton-X and Adenosine 5′-triphosphate Disodium (ATP) Salt were purchased from *Fisher Scientific* (Fair Lawn, NJ) and paraformaldehyde (PFA) from *Electron Microscopy Services* (Hatfield, PA). Propidium iodide (PI) from obtained from *Biotium* (Fremont, CA), forskolin from *BioVision* (Milpitas, CA), verapamil (Ver) from *Sigma-Aldrich* (Milwaukee, WI), tolvaptan (Tvp) from *AK Scientific* (Union City, CA) and arginine vasopressin (AVP) was purchased from *Bachem* (Torrance, CA). Laemmli 2X sample buffer was obtained from *BioRad* (Hercules, CA), cOmplete Protease Inhibitor Cocktail from *Roche* (St. Louis, MO) and Western blot visualization kit was obtained from *Thermo Scientific* (Rockford, IL). Nonfat dry milk was purchased from *LabScientific* (Livingston, NJ). Primary antibodies, acetylated-α-tubulin was acquired from *Abcam* (Cambridge, MA), β-actin from *Cell Biolabs* (San Diego, CA) and V2R antibody from *EMD Millipore* (Billerca, MA). Previously validated adenylyl cyclase 2, 3, 4, 5/6, 7, 8 and 9 antibodies were obtained from *Santa Cruz Biotech* (Santa Cruz, CA)^80^. The secondary antibodies, fluorescein anti-mouse, texas-red anti-rabbit and mounting media with DAPI were purchased from *Vector Laboratories* (Burlingame, CA).

### Cell culture

Porcine renal epithelial cells from proximal tubule (LL-CPK1), dog epithelial cells from inner medullary collecting duct (IMCD), and mouse vascular endothelial (ET) cells were cultured to a confluent monolayer in DMEM supplemented with 10% FBS at 37 °C in 5% CO2. In some experiments, LL-CPK1 cells were also grown on Corning Transwell permeable supports to induce polarization for receptor localization studies and allow antibody access to the basal membrane. ET and LL-CPK1 cells have been previously described in detail^5,81^. Once differentiated, different concentrations of tolvaptan or vasopressin was added to culture plates. Concentration for tolvaptan was determined to be 0.1 μM based on the optimal ciliary length increase based on dose-response studies, whereas a vasopressin concentration of 10 μM was selected because it maximally increases cAMP levels^82^. Verapamil was added to cells at a final concentration of 2 μM for 10 minutes before drug treatment^83^. The drugs were mixed in starvation medium (DMEM with 2% FBS) and cells were incubated for another 20 hours. Vehicle alone (PBS containing 0.0005% DMSO) was used as a control groups to account for the DMSO concentration in both tolvaptan and vasopressin working solutions.

### FACS analysis

Florescent-activated cell sorting (FACS) was used to investigate a possibility of an effect on cell division by tolvaptan (0.1 μM). Cells were harvested with and without drug treatment. Cells were then fixed using 70% ethanol and incubated with propidium iodide (PI), a DNA-intercalating fluorescent molecule, for 30 minutes at 37 °C. Cell division analysis was carried out with flow cytometry BDFacsverse with BD FACsuite software.

### Immunofluorescent staining

Cells were fixed for 10 minutes (4% PFA/2% sucrose in PBS) and permeabilized for 5 minutes (10% Triton X-100). Acetylated α-tubulin (1:10,000 dilution in PBS) and fluorescein isothiocyanate (FITC)-conjugated secondary (1:1000 dilution in PBS) antibodies were each incubated with the cells for 1 hour at 37 °C. For V2R visualization, V2R antibody (1:10,000 dilution in PBS) and texas-red conjugated anti-rabbit antibody were applied for 1 hour each at 37 °C. All adenylyl cyclase (AC) antibodies were used at a 1:1,000 dilution, and incubated with appropriate texas-red conjugated secondary antibody for 1 hour each at 37 °C. Slides were then mounted with dapi hard set mounting media (*VectorLabs*, Burlingame, CA). Nikon Eclipse Ti-E inverted microscope or Nikon Confocal microscope with NIS-Elements imaging software (version 4.30) was used to capture images of primary cilia. Automated image acquisition was conducted in large image scan mode at 100x magnification and Z-stacks of 0.1 µm thickness. All images are shown as maximum intensity projections except confocal generated images or stated otherwise.

### Flow-induced cytosolic calcium imaging

Cells were grown on glass-bottom plates. After treatment with the appropriate drug, the cells were incubated with 5 µM Fura-2 AM (*TEFLabs*, Austin, TX) at 37 °C for 30 minutes. After washing with DPBS, the cells were observed under a 40 × objective lens with a Nikon Eclipse Ti-E microscope. Fura-2 fluorescence images at excitation of 340/380 nm and emission of 510 nm were recorded. After equilibration under the microscope for 20 minutes, baseline calcium was recorded for 2 minutes and experimental data were acquired. Fluid-shear stress was then applied to cells with an Instech P720 peristaltic pump. The perfused fluid was pumped into the cell culture dish and retained at a shear stress of 1 dyne/cm^2^ (for epithelial cells) or 8 dyne/cm^2^ (for endothelial cells) with a constant flow rate of about 20 or 160 μl/sec, respectively. At the end of each experiment, the maximum calcium signal was obtained by perfusion of ATP (10 µM) to confirm cell viability. In addition to autofluorescence, both minimum and maximum fluorescence was collected as previously described^84^. Conditions for all experiments were maintained at 37 °C and 5% CO_2_ in a stage top cage incubator (okoLab, Burlingame, CA). Calcium analysis was then followed a standard calculation as previously described^84^.

### Generation of V2R-knockdown in renal epithelial LL-CPK1 cells

Arginine vasopressin receptor type-2 (AVPR2 or V2R) targeting shRNA construct (5′-ATC GCC TTG ATG GTG TTT GTG GCA CCT GC-3′: pAVP2Ra-C-shLenti) in a lentivirus backbone vector (TL513450) was ordered from Origene. Viral particles were generated in HEK cells using shRNA lentiviral packaging kit (TR30022) and passed through a 0.45-micron filter to remove cell debris. For transduction, epithelial cells were incubated with the collected viral particles and Polybrene (8 ug/ml; EMD Millipore).

For Western blot, total cell lysate was analyzed by SDS-PAGE on a 10% SDS-polyacrylamide gel. After separation, a semi-dry transfer was done using Bio-Rad system. The anti-V2R (1:1000 dilution) and anti-β-actin (1:500 dilution) antibodies were incubated with the PVDF (Polyvinylidene difluoride) membrane. Each incubation was done at 4 °C overnight. For visualization, horse radish peroxidase (HRP)-conjugated secondary antibodies anti-mouse (1:1000 dilution) or anti-rabbit (1:1000 dilution) were used. For dot blot experiment, cell or ciliary proteins were extracted in RIPA buffer with protease inhibitor. A 3 µl of sample extract was spotted onto a nitrocellulose membrane. After drying the membrane, it was blocked in 5% BSA (bovine serum albumin; *Promega*) for 1 hour. Incubation with V2R antibody (1:1000 dilution) was done for 30 minutes at room temperature. The membrane was then incubated with an HRP conjugated anti-rabbit antibody for 30 minutes at room temperature. For both Western and dot blots, the membrane was incubated with ECL (Enhanced Chemiluminescent; *Thermo Scientific*) for 1 minute. Visualization of the protein signal was done in Bio-Rad Image analysis and imager.

### Cilia isolation

Cells were plated on 100 mm dishes (6 for each cell line) with 10% FBS supplemented DMEM at 37 °C in 5% CO_2_. Cells were grown for 7 days and were starved overnight for differentiation as outlined above in cell culture methods. Cells were rinsed gently with PBS for a brief period and 10 ml of PBS was added. The dish was then placed on a rotary shaker (*ThermoFisher Scientific* MaxQ 2508) and shaken for 4 minutes at 360 rpm, resulting in a shear stress of 10 dyn/cm^2^. PBS was collected and transferred to a 50 ml centrifuge tube and spun for 10 minutes at 1,000 × g at 4 °C. After discarding the pellet, the supernatant was spun down in an ultracentrifuge (*ThermoFisher Scientific* Sorvall WX 100 + Ultracentrifuge) with a fixed angle rotor (*ThermoFisher Scientific*, T-647.5) at 40,000 × g for 45 minutes at 4 °C. After discarding the supernatant, the remaining pellet containing primary cilia, was resuspended in either RIPA buffer or resuspension buffer as previously discussed^43,44^.

### cAMP imaging and quantification

Intracellular cyclic AMP (cAMP) was quantified using ELISA and live-cell imaging. The cAMP ELISA Kit from *Cayman Chemical* (Cat. No. 581001) was used to measure total cAMP from cell population. Samples were lysed according to manufactural instructions in a 96-well plate format. After primary incubation for 18 hours, the plate was developed and read at 415 nm on a spectrophotometer.

For cAMP live-cell imaging, we tested both pc3.1-SSTR3-mICNBD-FRET and 5HT6-mCherry-cADDis. The generation of pc3.1-SSTR3-mICNBD-FRET and 5HT6-mCherry-cADDis have been previously described^85,86^. While both constructs worked fine in response to forskolin, we chose to use 5HT6-mCherry-cADDis for the rest of our studies to obtain a relatively better transfection in our cell line. Of note is that pc3.1-SSTR3-mICNBD-FRET was introduced to the cells with chemical transfection (jetPRIME; *Polyplus*), whereas Baculovirus transduction was utilized to express 5HT6-mCherry-cADDis (#D0211G; *Montana Molecular*). 2 × 10^5^ cells were plated in 6 well plates and grown in conditions described above for 3 days. Baculovius mediated transduction was then performed using a final concentration of 5 × 10^5^ VG (viral genes)/ml and 4 mM sodium butyrate. 12 hours after initiation of viral transduction media was replaced with starvation media and incubated for another 16 hours before imaging. To enable us to monitor cilia and cell body concurrently, we used side-view imaging as previously described^51^. After infection, baseline fluorescence was examined for each cell. Those cells expressing the cAMP sensor in the cilia were used in our studies. In addition to cilia specific cAMP sensor, a global cytosolic sensor (#U0200R; *Montana Molecular*) was also used to validate our imaging system.

Fluorescence readings were taken on individually for mCherry and cADDis (cAMP difference detector *in situ*) at excitation/emission wavelengths of 590/610 and 490/510 nm, respectively. A set of mCherry and cADDis images were captured at 0.8 frames per second. The mCherry signal was used to correct fluorescence artifact. Because cADDis is a downward cAMP reporter with a constant mCherry fluorescence, the mCherry/cADDis ratio therefore depicted intracellular cAMP level and was used to quantify changes in cilioplasmic and cytoplasmic cAMP. For ease of understanding, the cADDis signal was inverted to represent a direct relationship between cAMP level and cADDis intensity in our figures and movies (1/cADDis). Time-lapse recordings were acquired on NIS-Elements imaging software (version 4.30) and fluorescence intensities were measured for the duration of the experiment.

### Data analysis

All data are reported as a mean ± standard error of mean (s.e.m). Statistical analysis was performed using ANOVA (analysis of variance) followed by Bonferroni post-hoc test. Power analysis was determined from the coefficient variant. When our coefficient variant was above 15%, the number of experimental and corresponding control groups was increased. Because in all our studies both control and experimental groups were run in parallel, our control and experimental values represented matched number of observations. In some cases, all experimental groups (including the corresponding controls) were analyzed with the post-hoc test. In other cases, only selected pairs (control vs. experimental groups) were tested. Most of our statistical analyses were performed with *GraphPad* Prism v.7 software. In some cases, Microsoft Excel v.15.4 was used for regression analyses. Linear regression was performed to obtain a standard calibration curve and linear equation. In this case, the analysis was done with the ordinary least squares (OLS) regression of *y* on *x*. A non-linear logarithmic regression was used to fit the sigmoidal trend-curve to show dose-response relationship. Asterisks (*) denote statistically significant differences at various probability levels (*P*). The *P* values of the significant differences are indicated in the figure legends.

When image analyses were done on fixed-specimen, images were taken at different focal planes (*z-*stack). If needed, a 3-dimensional image was constructed from the XY planes to validate XZ and YZ field for completion of analysis or measurement. Automated image acquisition with a thickness of 0.1 µm were taken with Nikon A1R Confocal microscope and NIS-Elements imaging software v.4.30. This system was also used for image analyses (3D object reconstruction, image segmentation, etc.). For live-imaging, Nikon Eclipse Ti-E inverted microscope and Nikon NIS Element for Advanced Research software were used for image acquisition and analyses. Our live-imaging system with controlled environmental chamber allowed more flexibility for various excitation and emission spectra from a fast wavelength exchanger DG4/5 mirror reflection system. Images were not enlarged during image analysis to avoid a false empty magnification. Ciliary length measurements were done using the measurement tool in NIS-Elements imaging software on maximum intensity projections obtained from immunofluorescent images. All representative images and video frames are presented with scale bars to indicate the actual image reduction size at 0.8 frames per seconds (fps). Before Fura-2 experiments, a brightfield image focused on cell borders was obtained. Using intensity thresholds, a binary layer was created and overlaid with the Fura-2 images to create our representative images.

Free cytosolic calcium (Cyt Ca^2+^) concentrations were calculated with the formula [Cyt Ca^2+^ = Kd × [(R − Rmin)/(Rmax − R)] × (Fmax/Fmin), where Kd denotes the apparent dissociation constant of the Fura-2 indicator (145 nM), R is a ratio of 510 nm emission intensity with excitation at 340 and 380 nm, and Rmax and Rmin are fluorescence intensity ratios for the calcium-bound and calcium-unbound Fura-2 with excitation at 340 and 380, respectively. We determined the Rmax and Rmin values to be stable and independent of cell type. Fmax and Fmin were the fluorescence intensity values of Fura-2 with excitation at 380 nm under the same conditions. The calcium level was radiometrically calculated. Rmin and Rmax values denote the minimum and maximum radiometric signal ratios, respectively. At the end of each experiment, the minimum fluorescence (Rmin) was obtained by incubating the cells in calcium-free solution that contained 2 mM EGTA and 10 μM ionomycin at pH 8.6 to optimize the ionomycin effect. After the minimum signal ratio was determined, the cells were incubated with excess calcium (10 mM) to obtain the maximum signal ratio (Rmax). Signal intensities were collected from individual cells, as well as from the whole cell population/monolayer. All the fluorescence measurements were corrected for autofluorescence. Calcium analysis was then followed a standard calculation as previously described^84^.

## Supplementary information

Online Resource 1

Supplementary Movie 1

Supplementary Movie 2

Supplementary Movie 3

Supplementary Movie 4

## References

[1] Pazour GJ (2000). Chlamydomonas IFT88 and its mouse homologue, polycystic kidney disease gene tg737, are required for assembly of cilia and flagella. J. Cell Biol..

[2] Nauli SM (2006). Loss of Polycystin-1 in Human Cyst-Lining Epithelia Leads to Ciliary Dysfunction. J. Am. Soc. Nephrol..

[3] Vassilev PM (2001). Polycystin-2 is a novel cation channel implicated in defective intracellular Ca2+ homeostasis in polycystic kidney disease. Biochem. Biophys. Res. Commun..

[4] Xu C (2006). Human ADPKD primary cyst epithelial cells with a novel, single codon deletion in the PKD1 gene exhibit defective ciliary polycystin localization and loss of flow-induced Ca2+ signaling. AJP Ren. Physiol..

[5] Nauli SM (2008). Endothelial Cilia Are Fluid Shear Sensors That Regulate Calcium Signaling and Nitric Oxide Production Through Polycystin-1. Circulation.

[6] Nauli SM (2003). Polycystins 1 and 2 mediate mechanosensation in the primary cilium of kidney cells. Nat. Genet..

[7] Praetorius HA, Spring KR (2001). Bending the MDCK cell primary cilium increases intracellular calcium. J. Membr. Biol..

[8] Spasic M, Jacobs CR (2017). Lengthening primary cilia enhances cellular mechanosensitivity. Eur. Cell. Mater..

[9] Abdul-Majeed S, Nauli SM (2011). Dopamine receptor type 5 in the primary cilia has dual chemo- and mechano-sensory roles. Hypertens. (Dallas, Tex. 1979).

[10] Kathem SH (2014). Ciliotherapy: a novel intervention in polycystic kidney disease. J. Geriatr. Cardiol..

[11] Choi Y-H (2011). Polycystin-2 and phosphodiesterase 4C are components of a ciliary A-kinase anchoring protein complex that is disrupted in cystic kidney diseases. Proc. Natl. Acad. Sci. USA.

[12] Siroky BJ (2006). Loss of primary cilia results in deregulated and unabated apical calcium entry in ARPKD collecting duct cells. Am. J. Physiol. Renal Physiol..

[13] Pala R (2019). Personalized Nanotherapy by Specifically Targeting Cell Organelles To Improve Vascular Hypertension. Nano Lett..

[14] Pala Rajasekharreddy, Mohieldin Ashraf M., Sherpa Rinzhin T., Kathem Sarmed H., Shamloo Kiumars, Luan Zhongyue, Zhou Jing, Zheng Jian-Guo, Ahsan Amir, Nauli Surya M. (2019). Ciliotherapy: Remote Control of Primary Cilia Movement and Function by Magnetic Nanoparticles. ACS Nano.

[15] Grantham JJ, Ye M, Gattone VH, Sullivan LP (1995). *In vitro* fluid secretion by epithelium from polycystic kidneys. J. Clin. Invest..

[16] Ye M, Grantham JJ (1993). The Secretion of Fluid by Renal Cysts from Patients with Autosomal Dominant Polycystic Kidney Disease. N. Engl. J. Med..

[17] Mangoo-Karim R (1989). Renal epithelial fluid secretion and cyst growth: the role of cyclic AMP. FASEB J..

[18] Fenton RA, Brønd L, Nielsen S, Praetorius J (2007). Cellular and subcellular distribution of the type-2 vasopressin receptor in the kidney. Am. J. Physiol. Renal Physiol..

[19] Ausiello DA, Skorecki KL, Verkman AS, Bonventre JV (1987). Vasopressin signaling in kidney cells. Kidney Int..

[20] Nagao S (2006). Increased Water Intake Decreases Progression of Polycystic Kidney Disease in the PCK Rat. J. Am. Soc. Nephrol..

[21] Gattone VH, Wang X, Harris PC, Torres VE (2003). Inhibition of renal cystic disease development and progression by a vasopressin V2 receptor antagonist. Nat. Med..

[22] Gattone VH, Maser RL, Tian C, Rosenberg JM, Branden MG (1999). Developmental expression of urine concentration-associated genes and their altered expression in murine infantile-type polycystic kidney disease. Dev. Genet..

[23] Wang X, Wu Y, Ward CJ, Harris PC, Torres VE (2008). Vasopressin directly regulates cyst growth in polycystic kidney disease. J. Am. Soc. Nephrol..

[24] Reif GA (2011). Tolvaptan inhibits ERK-dependent cell proliferation, Cl^−^ secretion, and *in vitro* cyst growth of human ADPKD cells stimulated by vasopressin. Am. J. Physiol. Renal Physiol..

[25] Wang X, Gattone V, Harris PC, Torres VE (2005). Effectiveness of vasopressin V2 receptor antagonists OPC-31260 and OPC-41061 on polycystic kidney disease development in the PCK rat. J. Am. Soc. Nephrol..

[26] Lager DJ, Qian Q, Bengal RJ, Ishibashi M, Torres VE (2001). The pck rat: a new model that resembles human autosomal dominant polycystic kidney and liver disease. Kidney Int..

[27] Kim JK, Schrier RW (1998). Vasopressin processing defects in the Brattleboro rat: implications for hereditary central diabetes insipidus in humans?. Proc. Assoc. Am. Physicians.

[28] Torres VE (2016). Effect of Tolvaptan in Autosomal Dominant Polycystic Kidney Disease by CKD Stage: Results from the TEMPO 3:4 Trial. Clin. J. Am. Soc. Nephrol..

[29] Torres VE (2018). Multicenter, open-label, extension trial to evaluate the long-term efficacy and safety of early versus delayed treatment with tolvaptan in autosomal dominant polycystic kidney disease: the TEMPO 4:4 Trial. Nephrol. Dial. Transplant.

[30] Torres VE (2012). Tolvaptan in Patients with Autosomal Dominant Polycystic Kidney Disease. N. Engl. J. Med..

[31] Higashihara E (2011). Tolvaptan in autosomal dominant polycystic kidney disease: three years’ experience. Clin. J. Am. Soc. Nephrol..

[32] Torres VE (2017). Tolvaptan in Later-Stage Autosomal Dominant Polycystic Kidney Disease. N. Engl. J. Med..

[33] Cheng X, Ji Z, Tsalkova T, Mei F (2008). Epac and PKA: a tale of two intracellular cAMP receptors. Acta Biochim. Biophys. Sin. (Shanghai)..

[34] Besschetnova TY (2010). Identification of signaling pathways regulating primary cilium length and flow-mediated adaptation. Curr. Biol..

[35] Low SH (1998). Targeting of SNAP-23 and SNAP-25 in polarized epithelial cells. J. Biol. Chem..

[36] Abdul-Majeed S, Moloney BC, Nauli SM (2012). Mechanisms regulating cilia growth and cilia function in endothelial cells. Cell. Mol. Life Sci..

[37] Ou Y (2009). Adenylate cyclase regulates elongation of mammalian primary cilia. Exp. Cell Res..

[38] Sorokin S (1962). Centrioles and the formation of rudimentary cilia by fibroblasts and smooth muscle cells. J. Cell Biol..

[39] Rieder CL, Jensen CG, Jensen LCW (1979). The resorption of primary cilia during mitosis in a vertebrate (PtK1) cell line. J. Ultrastruct. Res..

[40] Ecelbarger CA, Chou CL, Lolait SJ, Knepper MA, DiGiovanni SR (1996). Evidence for dual signaling pathways for V2 vasopressin receptor in rat inner medullary collecting duct. Am. J. Physiol. Physiol..

[41] Faust, D. *et al*. Culturing primary rat inner medullary collecting duct cells. *J. Vis. Exp*., 10.3791/50366 (2013). 10.3791/50366 PMC3728753 23852264

[42] Nonoguchi H (1995). Immunohistochemical localization of V2 vasopressin receptor along the nephron and functional role of luminal V2 receptor in terminal inner medullary collecting ducts. J. Clin. Invest..

[43] Mohieldin AM (2015). Protein composition and movements of membrane swellings associated with primary cilia. Cell. Mol. Life Sci..

[44] Mitchell, K. A. P. Isolation of primary cilia by shear force. *Curr. Protoc. cell Biol*. **Chapter 3**, Unit 3.42.1-9 (2013). 10.1002/0471143030.cb0342s59 23728745

[45] Nielsen S., Chou C. L., Marples D., Christensen E. I., Kishore B. K., Knepper M. A. (1995). Vasopressin increases water permeability of kidney collecting duct by inducing translocation of aquaporin-CD water channels to plasma membrane. Proceedings of the National Academy of Sciences.

[46] Schlondorff D, Franki N (1980). Effect of vasopressin on cyclic AMP-dependent protein kinase in toad urinary bladder. Biochim. Biophys. Acta.

[47] Bishop GA, Berbari NF, Lewis J, Mykytyn K (2007). Type III adenylyl cyclase localizes to primary cilia throughout the adult mouse brain. J. Comp. Neurol..

[48] Wang Z (2009). Adult Type 3 Adenylyl Cyclase–Deficient Mice Are Obese. PLoS One.

[49] Raychowdhury MK (2009). Vasopressin receptor-mediated functional signaling pathway in primary cilia of renal epithelial cells. Am. J. Physiol. Renal Physiol..

[50] Wang Qian, Cobo-Stark Patricia, Patel Vishal, Somlo Stefan, Han Pyung-Lim, Igarashi Peter (2018). Adenylyl cyclase 5 deficiency reduces renal cyclic AMP and cyst growth in an orthologous mouse model of polycystic kidney disease. Kidney International.

[51] Jin X (2014). Cilioplasm is a cellular compartment for calcium signaling in response to mechanical and chemical stimuli. Cell. Mol. Life Sci..

[52] Yip K-P (2002). Coupling of vasopressin-induced intracellular Ca2+ mobilization and apical exocytosis in perfused rat kidney collecting duct. J. Physiol..

[53] Chou C-L (2004). Non-muscle myosin II and myosin light chain kinase are downstream targets for vasopressin signaling in the renal collecting duct. J. Biol. Chem..

[54] Brown D (2003). The ins and outs of aquaporin-2 trafficking. Am. J. Physiol. Renal Physiol..

[55] Verbalis JG (2011). Efficacy and safety of oral tolvaptan therapy in patients with the syndrome of inappropriate antidiuretic hormone secretion. Eur. J. Endocrinol..

[56] Schrier RW (2006). Tolvaptan, a Selective Oral Vasopressin V2 -Receptor Antagonist, for Hyponatremia. N. Engl. J. Med..

[57] Watkins PB (2015). Clinical Pattern of Tolvaptan-Associated Liver Injury in Subjects with Autosomal Dominant Polycystic Kidney Disease: Analysis of Clinical Trials Database. Drug Saf..

[58] U.S. Food and Drug Administration. Drug Approval Package: Jynarque (tolvaptan) Available at: https://www.accessdata.fda.gov/drugsatfda_docs/nda/2018/204441Orig1s000TOC.cfm. (Accessed: 19th December 2018) (2018).

[59] Muto S (2017). Long-term safety profile of tolvaptan in autosomal dominant polycystic kidney disease patients: TEMPO Extension Japan. Trial. Drug. Healthc. Patient Saf..

[60] Kuo IY (2014). Cyst formation following disruption of intracellular calcium signaling. Proc. Natl. Acad. Sci. USA.

[61] Lin F (2003). Kidney-specific inactivation of the KIF3A subunit of kinesin-II inhibits renal ciliogenesis and produces polycystic kidney disease. Proc. Natl. Acad. Sci. USA.

[62] Fischer E (2006). Defective planar cell polarity in polycystic kidney disease. Nat. Genet..

[63] Upadhyay VS (2014). Roles of dopamine receptor on chemosensory and mechanosensory primary cilia in renal epithelial cells. Front. Physiol..

[64] Lu T-L, Chang W-T, Chan C-H, Wu S-N (2019). Evidence for Effective Multiple K+ -Current Inhibitions by Tolvaptan, a Non-peptide Antagonist of Vasopressin V2 Receptor. Front. Pharmacol..

[65] Halls ML, Cooper DMF (2011). Regulation by Ca2+ -signaling pathways of adenylyl cyclases. Cold Spring Harb. Perspect. Biol..

[66] Wei J, Wayman G, Storm DR (1996). Phosphorylation and inhibition of type III adenylyl cyclase by calmodulin-dependent protein kinase II *in vivo*. J. Biol. Chem..

[67] Wei J (1998). Phosphorylation and inhibition of olfactory adenylyl cyclase by CaM kinase II in Neurons: a mechanism for attenuation of olfactory signals. Neuron.

[68] Otto EA (2005). Nephrocystin-5, a ciliary IQ domain protein, is mutated in Senior-Loken syndrome and interacts with RPGR and calmodulin. Nat. Genet..

[69] Rothschild SC, Francescatto L, Drummond IA, Tombes RM (2011). CaMK-II is a PKD2 target that promotes pronephric kidney development and stabilizes cilia. Development.

[70] Agarwal SR, Miyashiro K, Latt H, Ostrom RS, Harvey RD (2017). Compartmentalized cAMP responses to prostaglandin EP2 receptor activation in human airway smooth muscle cells. Br. J. Pharmacol..

[71] Wachten S (2010). Distinct pools of cAMP centre on different isoforms of adenylyl cyclase in pituitary-derived GH3B6 cells. J. Cell Sci..

[72] Agarwal SR (2014). Role of Membrane Microdomains in Compartmentation of cAMP Signaling. PLoS One.

[73] Dessauer CW (2009). Adenylyl cyclase–A-kinase anchoring protein complexes: the next dimension in cAMP signaling. Mol. Pharmacol..

[74] Hu Q, Nelson WJ (2011). Ciliary diffusion barrier: The gatekeeper for the primary cilium compartment. Cytoskeleton.

[75] Hsiao Y-C, Tuz K, Ferland RJ (2012). Trafficking in and to the primary cilium. Cilia.

[76] Takao D, Wang L, Boss A, Verhey KJ (2017). Protein Interaction Analysis Provides a Map of the Spatial and Temporal Organization of the Ciliary Gating Zone. Curr. Biol..

[77] Bachmann-Gagescu R (2015). The Ciliopathy Protein CC2D2A Associates with NINL and Functions in RAB8-MICAL3-Regulated Vesicle Trafficking. PLOS Genet..

[78] Garcia-Gonzalo FR (2011). A transition zone complex regulates mammalian ciliogenesis and ciliary membrane composition. Nat. Genet..

[79] Kwon RY, Temiyasathit S, Tummala P, Quah CC, Jacobs CR (2010). Primary cilium-dependent mechanosensing is mediated by adenylyl cyclase 6 and cyclic AMP in bone cells. FASEB J..

[80] Bogard AS, Xu C, Ostrom RS (2011). Human bronchial smooth muscle cells express adenylyl cyclase isoforms 2, 4, and 6 in distinct membrane microdomains. J. Pharmacol. Exp. Ther..

[81] Hull RN, Cherry WR, Weaver GW (1976). The origin and characteristics of a pig kidney cell strain, LLC-PK1. In Vitro.

[82] Tahara A (1998). Pharmacological characterization of the human vasopressin receptor subtypes stably expressed in Chinese hamster ovary cells. Br. J. Pharmacol..

[83] Jin X (2014). L-type calcium channel modulates cystic kidney phenotype. Biochim. Biophys. Acta.

[84] Nauli SM (2013). Non-Motile Primary Cilia as Fluid Shear Stress Mechanosensors. in. Methods in enzymology.

[85] Mukherjee S (2016). A novel biosensor to study cAMP dynamics in cilia and flagella. Elife.

[86] Tewson PH, Martinka S, Shaner NC, Hughes TE, Quinn AM (2016). New DAG and cAMP Sensors Optimized for Live-Cell Assays in Automated Laboratories. J. Biomol. Screen..

