# Author Correction: Sensory primary cilium is a responsive cAMP microdomain in renal epithelia

**DOI:** 10.1038/s41598-020-58754-5

**Published:** 2020-01-29

**Authors:** Rinzhin T. Sherpa, Ashraf M. Mohieldin, Rajasekharreddy Pala, Dagmar Wachten, Rennolds S. Ostrom, Surya M. Nauli

**Affiliations:** 1 Department of Biomedical & Pharmaceutical Sciences, Chapman University, Irvine, CA USA; 2 Institute of Innate Immunity, Department of Biophysical Imaging, University Hospital, University of Bonn, Bonn, Germany; 3 Minerva Max Planck Research Group, Molecular Physiology, Center of Advanced European Studies and Research, Bonn, Germany; 4 Department of Medicine, University of California Irvine, Irvine, CA USA

Correction to: *Scientific Reports* 10.1038/s41598-019-43002-2, published online 25 April 2019

The legend for Figure 1c is incomplete.

‘Time-lapse images represent the intracellular calcium level in response to fluid-shear stress (arrow) by epithelial and endothelial cells treated without (control, vehicle) and with tolvaptan (0.1 μM). Color bar indicates intracellular calcium level from low (black) to high (red). Corresponding brightfield images are shown in Supplementary Fig. S1.’

should read:

‘Time-lapse images represent the intracellular calcium level in response to fluid-shear stress (arrow) by epithelial and endothelial cells that were first treated with vehicle alone (control), and then treated with tolvaptan (0.1 μM) for 20 hours. Color bar indicates intracellular calcium level from low (black) to high (red). Corresponding brightfield images are shown in Supplementary Fig. S1.’

