# Supplementary material for: Sensory primary cilium is a responsive cAMP microdomain in renal epithelia
Source: Sci Rep. 2019 Apr 25;9:6523. doi: 10.1038/s41598-019-43002-2 (PMC6484033; doi:10.1038/s41598-019-43002-2)
Supplement: Supplementary file 1 — Online Resource 1 [file 41598_2019_43002_MOESM1_ESM.pdf]

Sensory primary cilium is a responsive cAMP microdomain in renal epithelia

Rinzhin Tshering Sherpa[1], Ashraf M. Mohieldin[1], Rajasekharreddy Pala[1], Dagmar Wachten[2,3], Rennolds S. Ostrom[1], Surya M. Nauli[1,4]

[1]Department of Biomedical & Pharmaceutical Sciences, Chapman University, Irvine, CA, USA
[2]Biophysical Imaging, Institute of Innate Immunity, University Hospital, University of Bonn, Germany
[3]Minerva Max Planck Research Group, Molecular Physiology, Center of Advanced European Studies and Research, Bonn, Germany
[4]Department of Medicine, University of California Irvine, Irvine, CA, USA

Corresponding Author:
Surya Nauli, Ph.D.
Chapman University
Harry and Diane Rinker Health Science Campus
9401 Jeronimo Road.
Irvine, CA 92618-1908
Tel: 714-516-5480
Fax: 714-516-5481
Email: nauli@chapman.edu; snauli@uci.edu

Supplementary Figure 1

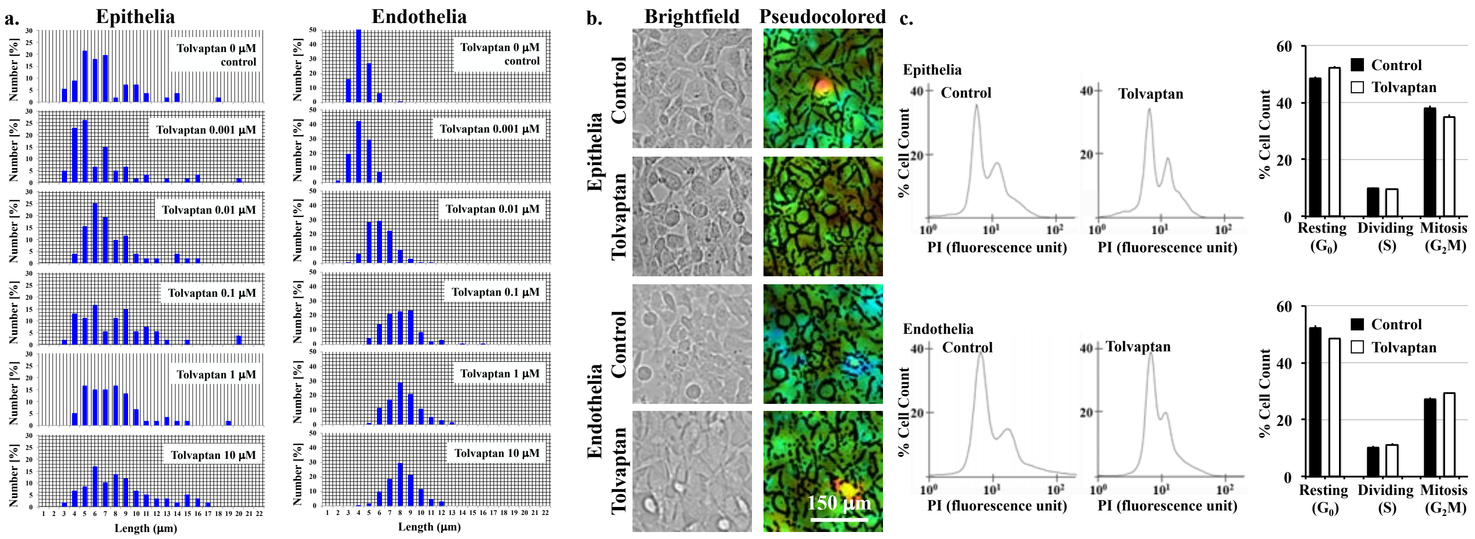

<u>**Supplementary Fig. 1** Tolvaptan increases ciliary length in the dose-dependent manner without affecting stages of cell division.</u>

a) Ciliary length distribution in renal epithelial LL-CPK1 cells without (vehicle control) and with tolvaptan (1 nM to 10 µM) treatment is shown in the histogram. Ciliary length (in µm) was plotted against the number of cells (in percent). 100 cells were randomly selected and measured from each preparation (N=3). **b)** Before Fura-2 experiments, a brightfield image was obtained (left panel). Intensity thresholds were applied to create a binary layer highlighting cell border. The binary layer was overlaid with the Fura-2 images to create our representative images (right panel). **c)** Flow cytometry analysis on cells without (vehicle control) and with tolvaptan (0.1 µM) treatment was conducted in endothelia and epithelia with propidium iodide (PI). Averaged cell counts in each cell division stage are shown in bar graph. N=3 in each group and treatment.

Supplementary Figure 2

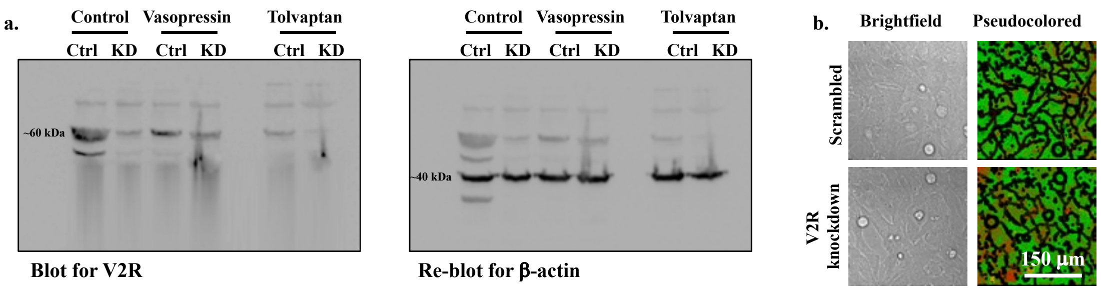

<u>Supplementary Fig. 2 V2R knockdown cells show lower expression of receptor.</u>

a) Using shRNA mediated silencing of strategy, V2R was knocked-down in LL-CPK1 cells (KD). The expression level of V2R was evaluated in response to vehicle (control), vasopressin (10 μM) and tolvaptan (0.1 μM) treatments in both scrambled control (Ctrl) and KD cells. Shown are full Western blot of V2R, in which the same membrane was reblotted for β-actin. **b)** A brightfield image was obtained (left panel) to represent cells used in Fura-2 experiments of both scrambled and V2R-knockdown cells. Baseline Fura-2 intensity is shown for both cell lines with a binary layer highlighting cell border (right panel).

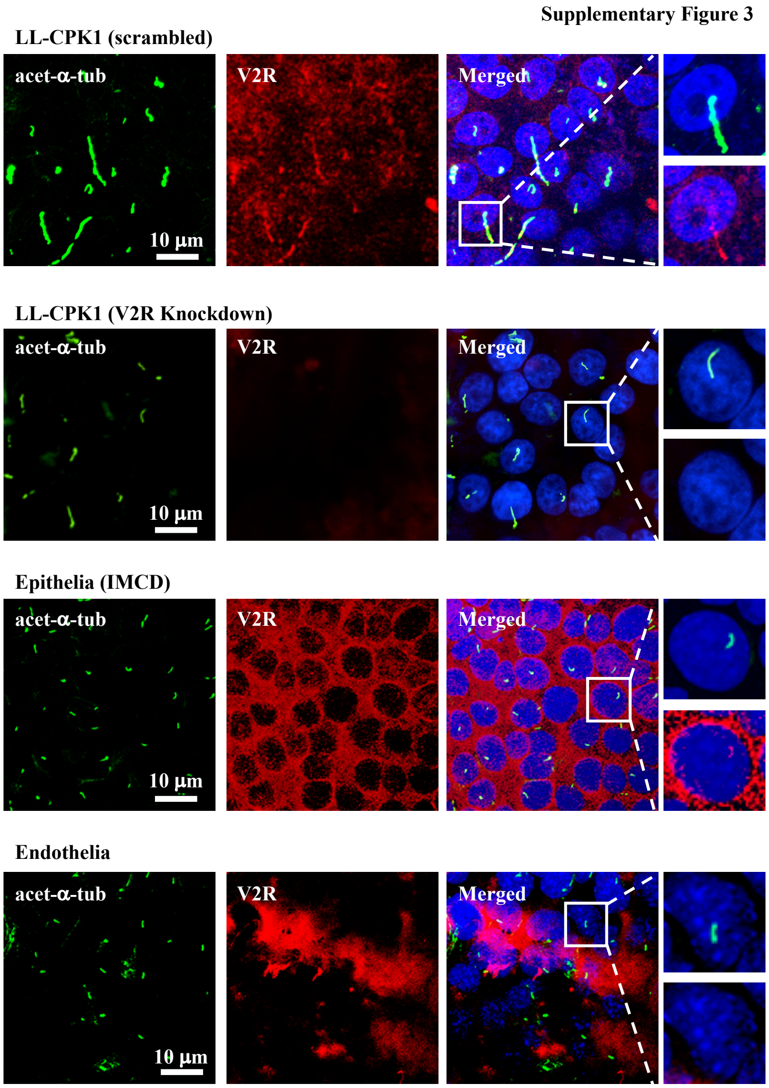

<u>Supplementary Fig. 3</u> <u>V2R is localized to primary cilia in renal epithelial cells.</u>

Renal epithelia from control LL-CPK1, V2R-knockdown LL-CPK1, IMCD and vascular endothelial cells were examined with ciliary marker acetylated-α-tubulin (acet-α-tub; green), V2R (red) and dapi (blue). Z-stack of confocal images at 0.1 µm slices were taken, and a maximum intensity projection was obtained. Magnified view of single cell showing V2R (red) localization in the cilium (green) are on the rightmost panel.

Supplementary Figure 4

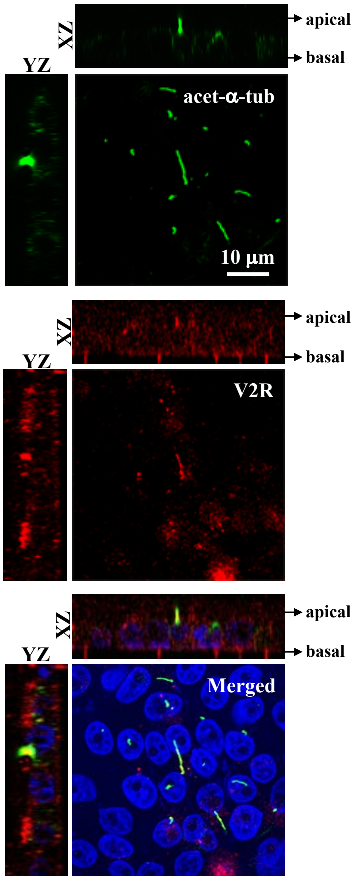

<u>**Supplementary Fig. 4** Apical and basolateral distribution of V2R in renal epithelial cells.</u> Polarized LL-CPK1 cells grown on permeable support were examined with ciliary marker acetylated-α-tubulin (acet-α-tub; green), V2R (red) and dapi (blue). Z-stack of confocal images at 0.1 µm increments were taken to create XZ and YZ axis plane. Apical and basal sides are labeled in the XZ view. The XY view shows a single focused plane of the cilia from the z-stack.

Supplementary Figure 5

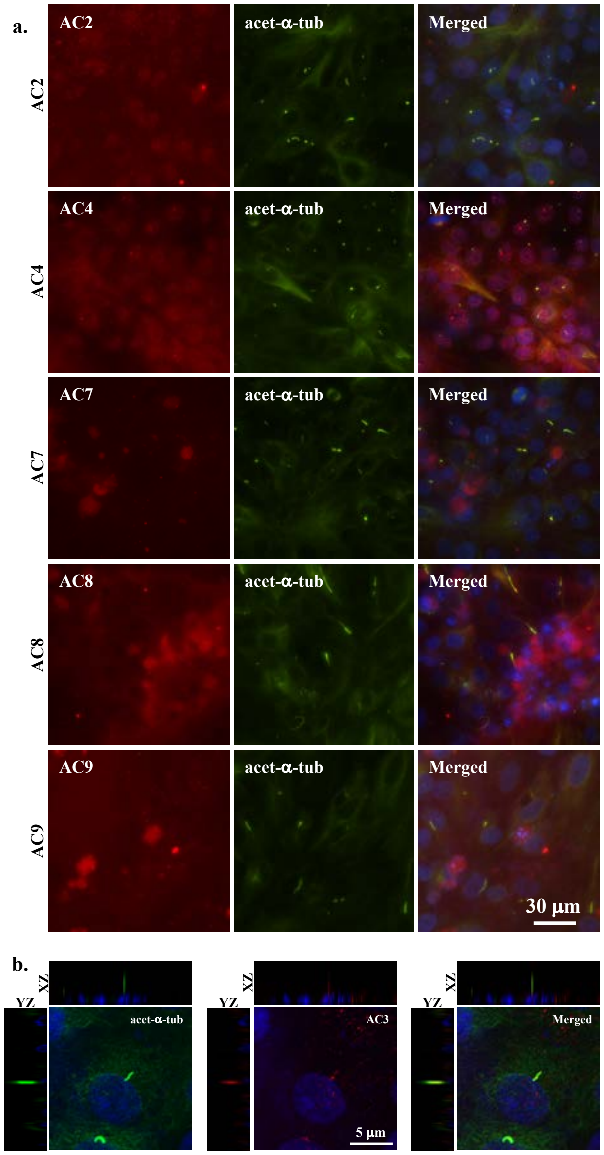

Supplementary Fig. 5 Among adenylyl cyclase (AC) isoforms-2, 3, 4, 7, 8 and 9 only AC3 is localized to the primary cilia of renal LL-CPK1 cells.

a) AC isoforms were examined for their ciliary immuno-localization. The first column of images shows AC isoforms (red) followed by ciliary marker acetylated-α-tubulin (acet-α-tub; green) and nucleus marker (merged; blue). **b)** LL-CPK1 cells were examined with AC3 (red), ciliary marker acetylated-α-tubulin (acet-α-tub; green), and dapi (blue). Z-stack images at 0.1 µm slices were taken and compiled to construct a 2D overlay, XZ and YZ axis views.

Supplementary Figure 6

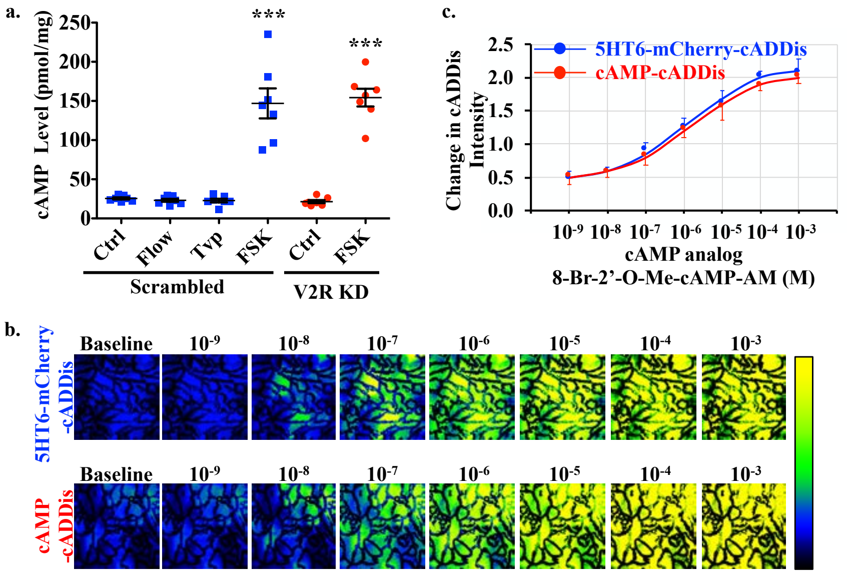

Supplementary Fig. 6 Global cAMP levels were measured using colorimetric competitive immunoassay, and 5HT6-mCherry-cADDis and cAMP-cADDis show similar dose response profiles.

a) Cell lysates collected from scrambled control and V2R-knockdown populations were used for cAMP quantification under different treatments (0.1 µM tolvaptan or Tvp; 10 µM vasopressin or AVP). Vehicle control with PBS (containing 0.005% DMSO) was used as a basal cAMP level. Fluid-shear stress was induced with 1.0 dyne/cm^2 for 5 minutes prior to the lysate collection. N=7 for each group. ***; $p<0.001$ compared to basal cAMP level. After expression of either 5HT6-mCherry-cADDis or cAMP-cADDis was confirmed in LL-CPK1 cells, cells were treated with adenylate cyclase inhibitor SQ22536 (10 µM) for 24 hours to deplete intracellular cAMP. The cAMP reporter profiles were examined with different concentrations of cell permeable cAMP analog 8-Br-2'-O-Me-cAMP-AM (1 nM to 1 mM; 10^{-9} to 10^{-3} M). Changes in intracellular cAMP concentration are shown in **b)** pseudocolored images after background subtraction and used to generate **c)** dose-response curves. N=3 for each group.

Supplementary Figure 7

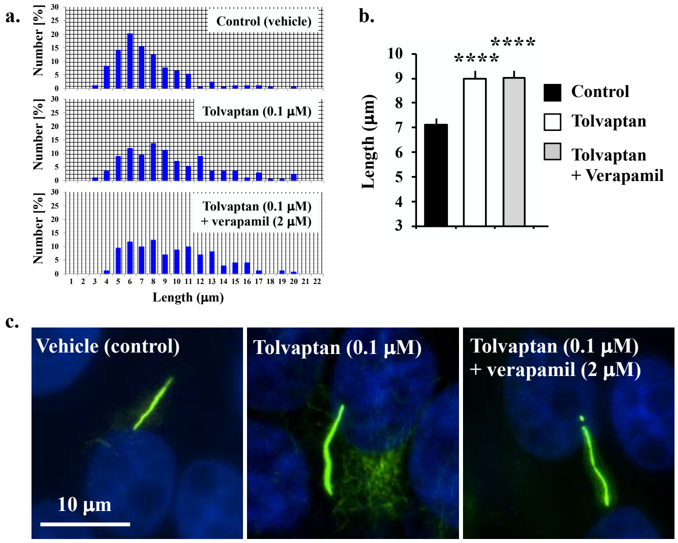

Supplementary Fig. 7 Calcium channel blocker, verapamil, does not affect increase in ciliary length induced by tolvaptan treatment.

a) Ciliary length distribution (in µm) in response to vehicle (control) or tolvaptan (0.1 µM) after preincubation for 10 min in vehicle or verapamil (2 µM) was plotted against the number of cells (in percent). About 150 cells were randomly selected and measured from each preparation (N=3). **b)** Corresponding bar graphs show ciliary length averages. ****P<0.00001. **c)** Representative immunostaining images are shown where cilia are in green (acetylated-α-tubulin) and nucleus in blue.

Movie Legends

Supplementary Movie 1. Vasopressin increases cilioplasmic and cytoplasmic cAMP levels.

Vasopression (10 μM) was added to a single cell at 50 second timepoint. The cilioplasmic and cytoplasmic cAMP were measured using a cAMP fluorescence reporter (5HT6-mCherry-cADDis). A set of mCherry and cADDis images were captured at 0.8 frames per second. The mCherry/cADDis ratio depicted intracellular cAMP level. Color bar shows the cAMP level.

Supplementary Movie 2. Forskolin increases cilioplasmic and cytoplasmic cAMP levels.

Forskolin (5 μM) was added to a single cell at 50 second timepoint. The cilioplasmic and cytoplasmic cAMP were measured using a cAMP fluorescence reporter (5HT6-mCherry-cADDis). A set of mCherry and cADDis images were captured at 0.8 frames per second. The mCherry/cADDis ratio depicted intracellular cAMP level. Color bar shows the cAMP level.

Supplementary Movie 3. In V2R-knockdown cell, forskolin increases cilioplasmic and cytoplasmic cAMP levels.

Forskolin (5 μM) was added to a V2R-knockdown cell at 50 second timepoint. The cilioplasmic and cytoplasmic cAMP were measured using a cAMP fluorescence reporter (5HT6-mCherry-cADDis). A set of mCherry and cADDis images were captured at 0.8 frames per second. The mCherry/cADDis ratio depicted intracellular cAMP level. Color bar shows the cAMP level.

<u>**Supplementary Movie 4.** A cilium undergoing flow-induced bending shows a decrease in ciliary cAMP levels.</u>

A single cell was challenged with a shear-stress of 1.0 dyn/cm^2 at 50 second timepoint. The cilioplasmic and cytoplasmic cAMP were measured using a cAMP fluorescence reporter (5HT6-mCherry-cADDis). A set of mCherry and cADDis images were captured at 0.8 frames per second. The mCherry/cADDis ratio depicted intracellular cAMP level. Color bar shows the cAMP level.
